# Genome-wide identification and transcriptome profiling expression analysis of the *U-box* E3 ubiquitin ligase gene family related to abiotic stress in maize (*Zea mays *L.)

**DOI:** 10.1186/s12864-024-10040-8

**Published:** 2024-02-01

**Authors:** Yongle Liu, Changgen Li, Aokang Qin, Wenli Deng, Rongrong Chen, Hongyang Yu, Yihua Wang, Jianbo Song, Liming Zeng

**Affiliations:** 1 College of Bioscience and Bioengineering, Jiangxi Agricultural University, Nanchang, 330045 People’s Republic of China; 2 College of Life Sciences, Nanjing University, Nanjing, 210095 People’s Republic of China; 3 Jiangxi Provincial Key Laboratory of Conservation Biology, Jiangxi Agricultural University, Nanchang, 330045 People’s Republic of China

**Keywords:** U-box, Abiotic stress, Gene expression, RNA-seq, Subcellular localization

## Abstract

**Background:**

The *U-box* gene family encodes E3 ubiquitin ligases involved in plant hormone signaling pathways and abiotic stress responses. However, there has yet to be a comprehensive analysis of the *U-box* gene family in maize (*Zea mays* L.) and its responses to abiotic stress.

**Results:**

In this study, 85 U-box family proteins were identified in maize and were classified into four subfamilies based on phylogenetic analysis. In addition to the conserved U-box domain, we identified additional functional domains, including Pkinase, ARM, KAP and Tyr domains, by analyzing the conserved motifs and gene structures. Chromosomal localization and collinearity analysis revealed that gene duplications may have contributed to the expansion and evolution of the *U-box* gene family. GO annotation and KEGG pathway enrichment analysis identified a total of 105 GO terms and 21 KEGG pathways that were notably enriched, including ubiquitin-protein transferase activity, ubiquitin conjugating enzyme activity and ubiquitin-mediated proteolysis pathway. Tissue expression analysis showed that some *ZmPUB* genes were specifically expressed in certain tissues and that this could be due to their functions. In addition, RNA-seq data for maize seedlings under salt stress revealed 16 stress-inducible plant *U-box* genes, of which 10 genes were upregulated and 6 genes were downregulated. The qRT-PCR results for genes responding to abiotic stress were consistent with the transcriptome analysis. Among them, *ZmPUB13*, *ZmPUB18*, *ZmPUB19* and *ZmPUB68* were upregulated under all three abiotic stress conditions. Subcellular localization analysis showed that *ZmPUB19* and *ZmPUB59* were located in the nucleus.

**Conclusions:**

Overall, our study provides a comprehensive analysis of the *U-box* gene family in maize and its responses to abiotic stress, suggesting that *U-box* genes play an important role in the stress response and providing insights into the regulatory mechanisms underlying the response to abiotic stress in maize.

**Supplementary Information:**

The online version contains supplementary material available at 10.1186/s12864-024-10040-8.

## Background

Plant growth is frequently challenged by various abiotic stresses, including salt, drought, cold and heat, exerting a substantial negative impact on global crop productivity. It is estimated that up to 50% of global crop losses can be attributed to abiotic stress, with more than 90% of these losses due to salt, drought and temperature stress [1]. Amidst the intricate molecular responses to abiotic stress, protein ubiquitination has become a focal point of investigation due to its dynamic alterations in response to stress stimuli [2].

The ubiquitin–proteasome pathway is one of the major protein degradation pathways in nuclear organisms [3]. In the ubiquitination pathway, the binding of ubiquitin to the target protein is mediated by a three-step enzyme cascade. The protein is catalyzed by the ubiquitin-activating enzyme E1, ubiquitin-binding enzyme E2s and ubiquitin ligase E3s [4, 5], and the protein can be recognized and degraded by the proteasome after labeling with polyubiquitin. E3 ubiquitin ligases are the largest family in ubiquitin proteasome degradation mechanisms, which include diverse types such as the RING, F-box, HECT and U-box [6, 7]. Studies have shown that RING and U-box type E3s are widely involved in abiotic stress processes, while the F-box protein is the core component of SKP1-Cullin1-F-box protein type E3 and plays an important role in the abiotic stress response [8]. The U-box protein structure, which functions as a ubiquitin ligase, is a conserved motif comprising approximately 70 amino acids. It is prevalent in fungi, plants, and animals, and it frequently interacts with ARM repeats in plants. This interaction influences protein–protein interactions and plays a crucial role in signal transduction cascades [9].

Crucially, E3 ubiquitin ligases, particularly U-box proteins, have been identified as key regulators enhancing abiotic stress tolerance in major food crops. For instance, in rice, *OsPUB15* acts as a negative regulator of cell death and abiotic stress responses, and its overexpression enhances salt tolerance in plants [10]. Moreover, *OsPUB41* plays a crucial role as a negative regulator in modulating drought stress tolerance [11]. Similarly, U-box proteins in wheat, including TaPUB1 and TaPUB15, exert positive effects on salt stress tolerance [12, 13], while *TaPUB26* acts as a negative regulator in response to salt stress [14] In maize, *ZmAIRP4* enhances drought stress tolerance [15], and *ZmRFP1* responds to drought stress in an ABA-dependent manner [16]. These examples underscore the intricate involvement of U-box ubiquitin ligases in the modulation of abiotic stress responses across diverse plant species. Furthermore, U-box ubiquitin ligases associated with abiotic stress have been identified in various plant species, including soybean, tomato, potato, pepper, apple and *Arabidopsis,* where they play crucial roles in regulating drought tolerance, salt tolerance and ABA signaling [17–22].

The *U-box* gene family has been identified in numerous plants, and the number of genes varies among species. In crop plants, *U-box* genes have been identified in the genomes of various plants, including rice, cabbage, soybean, tomato, barley, cotton (*G. barbadense*), wheat, sorghum, potato, *Brassica oleracea* L., wild emmer wheat and *Solanum tuberosum*, with a total of 41 to 213 genes, respectively [23–34]. In fruit plants such as banana, stony hard peach, white pear, apple and strawberry, with a total of 62 to 765 *U-box* genes have been identified, respectively [35–39].

Despite the important role of E3 ubiquitin ligase in abiotic stress responses in model plants such as *Arabidopsis*, rice and wheat, the understanding of their involvement in maize remains limited. Maize, one of the most widely grown crops in the world, often faces challenges such as salinity, drought, cold and heat stress. Drought alone inflicts an annual economic impact of $20 billion on maize yields [15]. Consequently, enhancing the stress resistance of maize plants is crucial for achieving higher yields. Despite numerous individual maize genes have been analyzed, a comprehensive study of the maize genome in response to abiotic stress is currently lacking. Therefore, it is essential to explore the role of *U-box* genes in stress signaling and to clarify their contribution to maize growth and development. These studies will provide a theoretical foundation for comprehending stress response mechanisms and provide a basis for selecting and breeding improved maize varieties with enhanced tolerance. The insights derived from this study promise a deeper understanding of the functions of the maize *U-box* gene family, paving the way for advancements in crop resilience and agricultural productivity.

## Results

### Identification of *ZmPUB* family genes in maize

To identify *U-box* family genes, we used the U-box domain (PF04564) to perform bioinformatic searches in the maize genome database using HMMER 3.0. The accuracy of the candidate *U-box* genes was checked using the Pfam database with an E-value cutoff level of 1.0. A total of 85 *ZmPUB* genes were confirmed and renamed *ZmPUB1*-*85* based on the order of their gene IDs (Table 1). The molecular weights of the 85 ZmPUB proteins ranged from 10 to 150 kDa, and the predicted amino acid quantities ranged from 90 to 1400. Among the 85 ZmPUB proteins, 57 ZmPUB proteins were acidic proteins with theoretical isoelectric points ranging from 4.65 to 6.97. There were 21 ZmPUB proteins with theoretical isoelectric points between 8.12 and 9.36 and are basic proteins. The other 7 ZmPUB proteins were electroneutral, with theoretical isoelectric points ranging from 7.01 to 7.93. The amino acid length and protein characteristics of these proteins were evidently different, suggesting that ZmPUB family members have different characteristics and play different roles in biological processes. Subcellular localization prediction results showed that most of the genes were located in the nucleus, and a few genes were predicted to be located in both the cytoplasm and the nucleus.

**Table 1:** Basic information of *U-box* family genes in maize

S. No	Gene ID	Accession number	Other domain	Predicted protein (aa)	Mol wt (kDa)	Pl	Chromosome	Putative localization
1	*ZmPUB1*	GRMZM2G000540_P01	zf-CCHC 2 DWNN	829	92.12234	8.82	1	Nucleus
2	*ZmPUB2*	GRMZM2G007486_P01	Unknown	126	14.51222	8.85	1	Nucleus
3	*ZmPUB3*	GRMZM2G010488_P01	Pkinase	862	95.13482	6.06	4	Nucleus
4	*ZmPUB4*	GRMZM2G013776_P01	Unknown	392	41.73596	6.62	2	Nucleus
5	*ZmPUB5*	GRMZM2G017852_P01	Arm	825	89.50287	5.38	4	Nucleus
6	*ZmPUB6*	GRMZM2G018059_P01	Pkinase Tyr Usp	827	93.75523	6.56	2	Chloroplast Cytoplasm Nucleus
7	*ZmPUB7*	GRMZM2G019777_P01	Arm	663	71.21743	5.81	8	Nucleus
8	*ZmPUB8*	GRMZM2G020196_P01	Arm	705	76.20938	8.95	4	Nucleus
9	*ZmPUB9*	GRMZM2G025037_P01	Arm	706	76.12597	5.93	4	Nucleus
10	*ZmPUB10*	GRMZM2G025214_P06	Unknown	278	31.70907	5.37	6	Nucleus
11	*ZmPUB11*	GRMZM2G027375_P01	Arm	663	71.02884	6.35	3	Nucleus
12	*ZmPUB12*	GRMZM2G030805_P01	Ufd2P core	1031	115.72181	5.25	1	Nucleus
13	*ZmPUB13*	GRMZM2G031624_P01	Unknown	343	35.94259	6.16	10	Nucleus
14	*ZmPUB14*	GRMZM2G033521_P01	Unknown	810	89.04951	5.53	3	Cytoplasm Nucleus
15	*ZmPUB15*	GRMZM2G037314_P01	DUF3475	531	59.22145	6.43	5	Nucleus
16	*ZmPUB16*	GRMZM2G037698_P01	Prp19 WD40	526	57.56017	6	1	Nucleus
17	*ZmPUB17*	GRMZM2G040385_P01	Prp19	91	10.23146	5.09	4	Nucleus
18	*ZmPUB18*	GRMZM2G041141_P01	Unknown	418	42.92928	7.62	5	Nucleus
19	*ZmPUB19*	GRMZM2G046848_P01	Pkinase	617	69.86194	8.26	1	Nucleus
20	*ZmPUB20*	GRMZM2G050734_P01	KAP	773	86.59611	5.4	5	Nucleus
21	*ZmPUB21*	GRMZM2G055052_P01	Unknown	447	47.26997	8.44	1	Nucleus
22	*ZmPUB22*	GRMZM2G057436_P01	Arm	872	94.364	5.83	6	Nucleus
23	*ZmPUB23*	GRMZM2G059042_P01	Unknown	444	45.68324	6.9	10	Chloroplast Nucleus
24	*ZmPUB24*	GRMZM2G062499_P01	Arm	465	50.26783	6.18	5	Nucleus
25	*ZmPUB25*	GRMZM2G063394_P01	Unknown	316	35.31218	5.42	10	Nucleus
26	*ZmPUB26*	GRMZM2G065612_P01	Unknown	1029	112.47641	6.01	9	Nucleus
27	*ZmPUB27*	GRMZM2G069070_P01	Unknown	444	46.9184	6.93	1	Nucleus
28	*ZmPUB28*	GRMZM2G071484_P01	Unknown	436	47.11529	8.27	5	Nucleus
29	*ZmPUB29*	GRMZM2G073310_P01	Unknown	452	46.41877	8.12	6	Nucleus
30	*ZmPUB30*	GRMZM2G075104_P01	Ufd2P core	1029	115.61575	5.33	9	Nucleus
31	*ZmPUB31*	GRMZM2G079284_P01	Unknown	149	16.48401	4.65	9	Nucleus
32	*ZmPUB32*	GRMZM2G092128_P01	Unknown	415	45.05753	7.93	2	Nucleus
33	*ZmPUB33*	GRMZM2G092550_P01	Pkinase	894	99.48393	6.93	7	Nucleus
34	*ZmPUB34*	GRMZM2G092652_P01	Arm	564	61.63181	5.77	1	Nucleus
35	*ZmPUB35*	GRMZM2G098128_P01	Unknown	448	47.76035	8.76	2	Nucleus
36	*ZmPUB36*	GRMZM2G099648_P01	Unknown	763	85.22459	6.1	9	Nucleus
37	*ZmPUB37*	GRMZM2G100090_P01	Arm	561	57.9277	7.01	7	Nucleus
38	*ZmPUB38*	GRMZM2G101754_P01	Pkinase	883	96.41423	5.95	4	Nucleus
39	*ZmPUB39*	GRMZM2G104769_P02	ANAPC3	275	30.93224	6.2	6	Nucleus
40	*ZmPUB40*	GRMZM2G115000_P01	Unknown	799	87.36612	5.83	3	Nucleus
41	*ZmPUB41*	GRMZM2G125034_P01	Arm	670	70.93965	6.97	6	Nucleus
42	*ZmPUB42*	GRMZM2G127524_P01	Unknown	967	107.17220	6.26	4	Nucleus
43	*ZmPUB43*	GRMZM2G127690_P01	Unknown	429	44.28904	8.6	4	Nucleus
44	*ZmPUB44*	GRMZM2G132671_P01	Prp19	133	15.31798	9.27	5	Nucleus
45	*ZmPUB45*	GRMZM2G135629_P01	Unknown	866	94.99933	5.41	10	Nucleus
46	*ZmPUB46*	GRMZM2G135713_P01	Unknown	408	43.3022	8.7	3	Nucleus
47	*ZmPUB47*	GRMZM2G136313_P01	Arm	645	71.01205	6.02	1	Nucleus
48	*ZmPUB48*	GRMZM2G150489_P01	Myb DNA-bind 3	424	48.41	5.88		Chloroplast Nucleus
49	*ZmPUB49*	GRMZM2G151204_P01	Unknown	452	48.42013	8.42	5	Nucleus
50	*ZmPUB50*	GRMZM2G152857_P01	Unknown	682	73.6754	5.5	10	Nucleus
51	*ZmPUB51*	GRMZM2G152919_P01	Unknown	463	49.45294	7.46	9	Nucleus
52	*ZmPUB52*	GRMZM2G153127_P01	WD40	1398	148.88145	5.78	3	Nucleus
53	*ZmPUB53*	GRMZM2G159247_P01	Pkinase Tyr	337	36.05656	9.36	4	Nucleus
54	*ZmPUB54*	GRMZM2G160304_P01	Unknown	248	26.37625	5.72	1	Chloroplast Nucleus
55	*ZmPUB55*	GRMZM2G160370_P01	Arm	729	78.93531	8.54	5	Nucleus
56	*ZmPUB56*	GRMZM2G168980_P01	Unknown	425	45.64891	5.49	5	Nucleus
57	*ZmPUB57*	GRMZM2G169690_P01	Unknown	409	44.06441	8.66	10	Nucleus
58	*ZmPUB58*	GRMZM2G301512_P01	Pkinase Usp	738	81.90228	5.55	4	Nucleus
59	*ZmPUB59*	GRMZM2G303964_P01	Unknown	429	44.35007	8.6	5	Nucleus
60	*ZmPUB60*	GRMZM2G304010_P01	Unknown	358	36.87724	6.41	10	Nucleus
61	*ZmPUB61*	GRMZM2G305822_P01	Pkinase	660	74.11796	6.2	9	Nucleus
62	*ZmPUB62*	GRMZM2G314412_P01	Unknown	980	106.34002	5.94	2	Nucleus
63	*ZmPUB63*	GRMZM2G315431_P01	Arm	698	73.95624	8.12	7	Cytoplasm Nucleus
64	*ZmPUB64*	GRMZM2G324540_P01	Prp19 WD40	526	57.64236	5.9	5	Nucleus
65	*ZmPUB65*	GRMZM2G331260_P01	Unknown	94	10.75643	7.78	7	Nucleus
66	*ZmPUB66*	GRMZM2G347102_P01	Arm	320	35.7955	5.72	3	Nucleus
67	*ZmPUB67*	GRMZM2G349344_P01	Pkinase	875	97.57628	5.8	2	Nucleus
68	*ZmPUB68*	GRMZM2G351387_P01	Arm	672	72.94428	5.57	3	Nucleus
69	*ZmPUB69*	GRMZM2G361100_P01	Unknown	400	41.64027	9	2	Nucleus
70	*ZmPUB70*	GRMZM2G373329_P01	Unknown	510	56.78856	5.61	1	Nucleus
71	*ZmPUB71*	GRMZM2G376085_P01	Arm	800	87.57675	5.79	3	Cytoplasm Nucleus
72	*ZmPUB72*	GRMZM2G389462_P01	Arm	641	71.00432	6.61	5	Nucleus
73	*ZmPUB73*	GRMZM2G389789_P01	Unknown	840	97.87358	5.55	2	Nucleus
74	*ZmPUB74*	GRMZM2G406758_P01	Unknown	261	27.91184	5.96	9	Nucleus
75	*ZmPUB75*	GRMZM2G425965_P01	Arm	533	54.68241	9.04	2	Nucleus
76	*ZmPUB76*	GRMZM2G433433_P01	Pkinase	757	83.21386	6.27	9	Nucleus
77	*ZmPUB77*	GRMZM2G452016_P01	Arm	630	68.16019	6.43	9	Nucleus
78	*ZmPUB78*	GRMZM2G471733_P01	Arm	638	68.72468	5.88		Nucleus
79	*ZmPUB79*	GRMZM2G475702_P01	Arm	694	73.54083	6.46	4	Nucleus
80	*ZmPUB80*	GRMZM2G476914_P01	Unknown	459	49.25054	7.02	1	Nucleus
81	*ZmPUB81*	GRMZM5G811633_P01	Unknown	409	42.28553	7.06	4	Chloroplast Nucleus
82	*ZmPUB82*	GRMZM5G821267_P01	Unknown	803	87.23282	5.61	8	Nucleus
83	*ZmPUB83*	GRMZM5G855994_P01	Unknown	442	45.70798	9.29	9	Nucleus
84	*ZmPUB84*	GRMZM5G891990_P01	Arm Neurochondrin	726	78.48884	5.10	1	Nucleus
85	*ZmPUB85*	GRMZM5G893055_P01	Unknown	573	59.10107	8.32	1	Nucleus

^a^ Basic information of *U-box* family genes in maize. Including other domain, predicted protein (aa), molecular weight (Mol wt), isoelectric point (Pl) and chromosome putative localization

### Phylogenetic relationship of PUB proteins in maize, rice and *Arabidopsis*

**Fig. 1:**
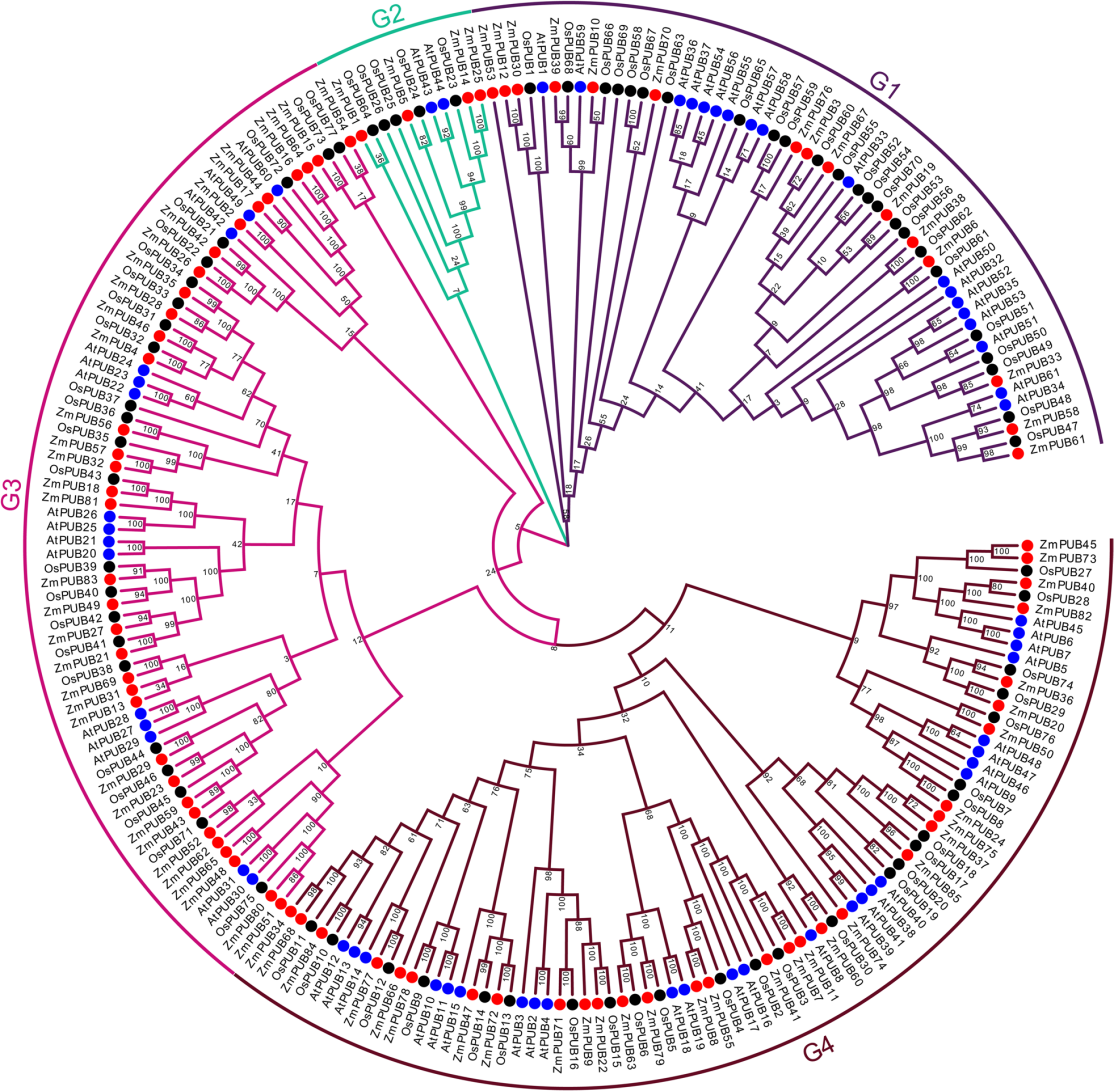
Evolutionary relationship analysis of the U-box protein family in maize, *Arabidopsis thaliana* and *Oryza sativa*. Phylogenetic tree of the *U-box* gene family in maize, *Arabidopsis* and rice. Multiple sequence alignments for U-box domain sequences of 85 ZmPUBs, 61 AtPUBs and 77 OsPUBs were conducted via MEGA 7.0. The phylogenetic tree was established using the neighbor-joining method with MEGA7.0 software, employing the p-distance method and a 1000-bootstrap value. The resulting *PUB* genes were categorized into four distinct groups (G1-G4), each identified by a unique color

To investigate the evolutionary relationships among *PUB* genes, we constructed a neighbor-joining (NJ) phylogenetic tree using U-box protein sequences from maize, rice and *Arabidopsis* (85 from maize, 77 from rice and 61 from *Arabidopsis*) (Table S1, Table S2 and Table S3). The topology of the phylogenetic tree was divided into four subfamilies. Notably, Group 4 exhibited the highest number of *PUB* genes, encompassing a total of 73, while Group 2 contained the fewest *PUB* genes, numbering only 11. Additionally, Group 1 and Group 3 consisted of 57 and 59 genes, respectively (Fig. 1).

Although the domains were not unequivocally categorized within the phylogenetic groups, it was evident that each group contained ZmPUBs, all of which featured the U-box domain. Notably, Group 4, the largest group, comprised 31 ZmPUBs, 25 OsPUBs and 17 AtPUBs and was distinguished by the presence of 20 ARM domains. The ZmPUB proteins within the phylogenetic tree were predominantly associated with Prp19 domain-containing proteins, primarily within the G3 subfamily. On the other hand, Pkinase, Pkinase Tyr, Ufd2P core and Usp domain-containing proteins were primarily found within the G1 subfamily (Fig. 1). These associations underscore the relationship between the phylogeny and evolution of ZmPUB proteins and suggest that the U-box protein domain has coevolved alongside other functional regions. In general, the *PUB* genes of maize and rice exhibited a closer evolutionary relationship to each other than to those of *Arabidopsis*.

### Functional domain analysis of the U-box proteins

**Fig. 2:**
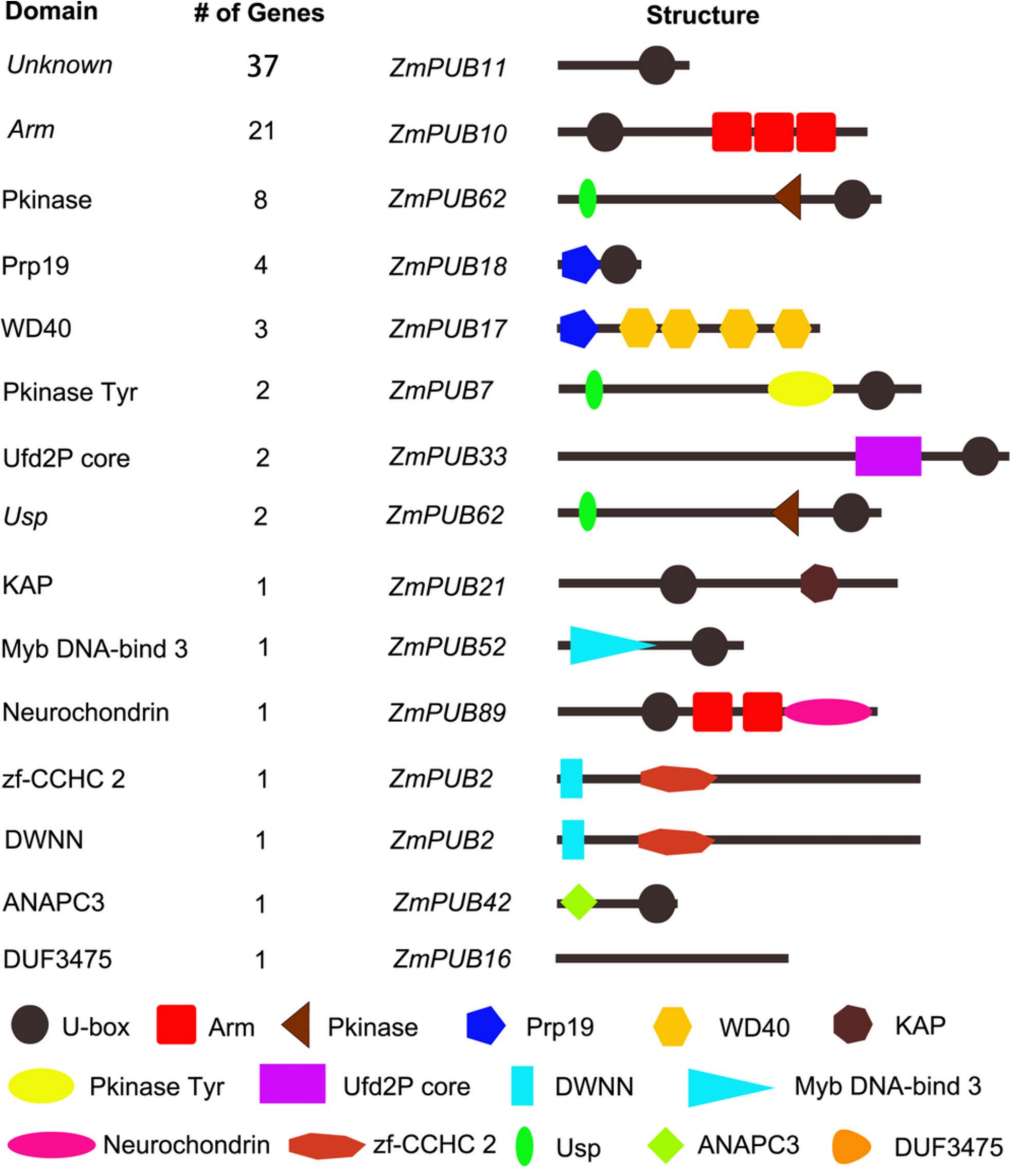
Number and domain structure of U-box proteins in maize. The domain names are taken from the Pfam database, and the position of the domain markers also refers to the position shown in the database. The different types of domains are marked with graphs of different colors and shapes, and a representative gene is provided for each domain. "Unknown" designates that, apart from the U-box domain, no other evident functional domains were identified

The SMART and Pfam database inquiries revealed that the U-box proteins contained several known or unknown conserved domains, which are essential for functionality and have been designated as functional domains (Fig. 2). Some U-box proteins had no other obvious interaction domains or had a few rare functionally uncertain domains, collectively categorized as 'Unknown' (only have U-box). Among the 85 U-box protein families, 37 genes had no other obvious functional domains except U-box (Unknown), 21 genes contain the Arm domain, 8 genes contain the Pkinase domain, and 4 genes contain the Prp19 domain. In addition, other functional domains were detected, such as WD40, Tyr, Ufd2P core, Usp, ANAPC3, DWNN, KAP, Myb DNA-bind3, Neurochondrin and zf-CCHC2. The ARM repeat is a series repeat motif approximately 40 amino acids long that binds to S-site receptor kinase (SRK) and is an important part of the downstream SRK signaling pathway [40]. Studies have shown that the U-box protein PHOR1 of potato (*Solanum tuberosum*) with these ARM repeat may play a role in gibberellin signaling [41]. These domains are essential for plant defense and disease resistance.

### Chromosome localization and collinearity analysis of *ZmPUBs*

**Fig. 3:**
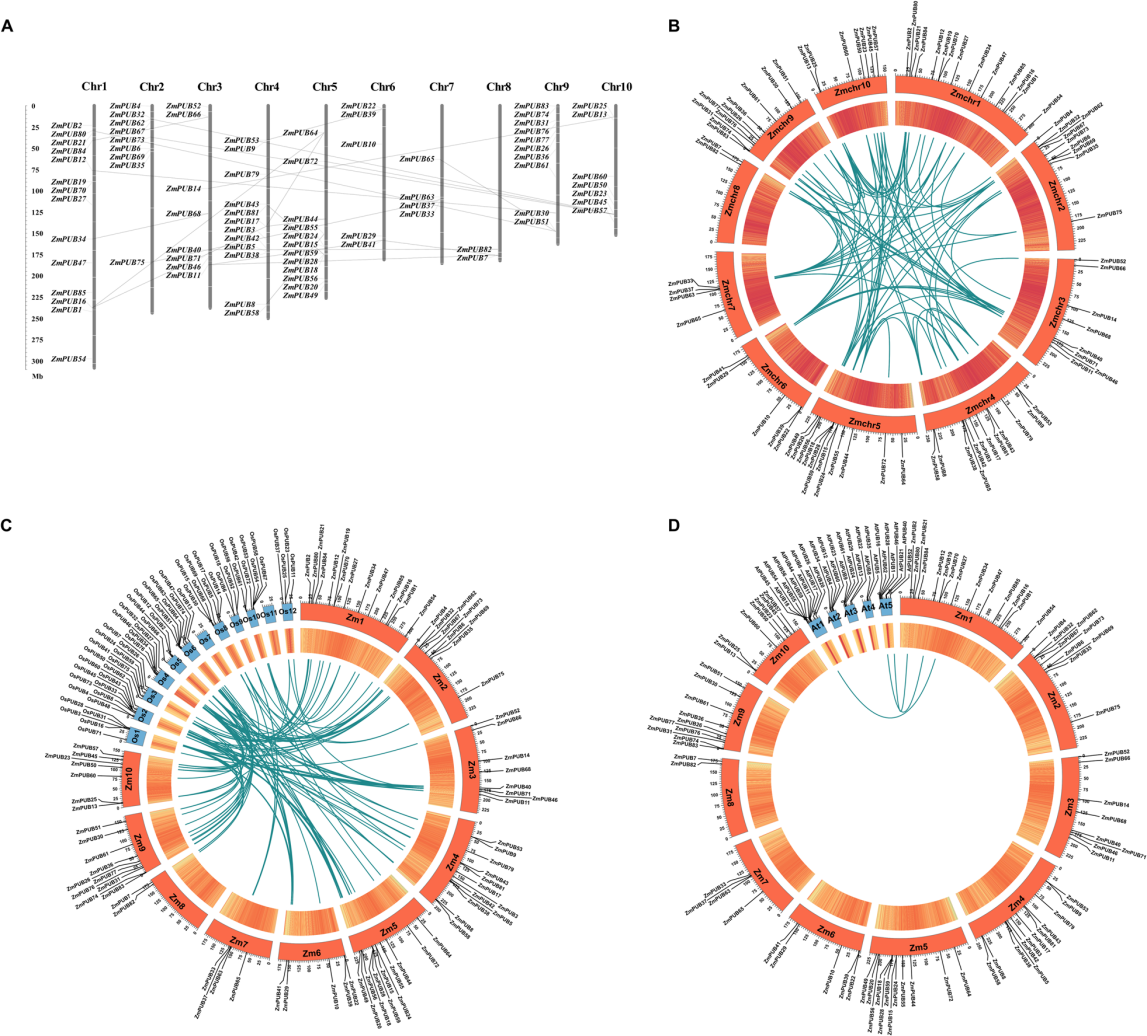
Chromosomal localizations and gene duplications of the *ZmPUB* family genes (A), collinearity analysis with *Arabidopsis* and rice (B-D). **A** The scale represents megabases (Mb), and the chromosome number is shown at the top of each bar. The protein sequences encoded by straight-line junction genes are more than 70% similar. **B** Collinearity relationships of *U-box* genes in maize. **C** Collinear relationships between *U-box* genes in maize and *Arabidopsis thaliana*. **D** Collinearity relationships between *U-box* genes in maize and rice. Blue lines represent collinear gene pairs of the *PUB* gene family. The inner circular heatmap represents gene density. The outer circles with scales represent chromosomes

According to the information on the physical positions of *ZmPUBs* in maizeGDB, the 85 *ZmPUB* genes, except for two genes that did not appear on the nucleus, were distributed randomly on the 10 chromosomes in maize, with the most genes on chromosome 1 and the fewest genes on chromosome 8 (Fig. 3A and Table S4). The results showed that *ZmPUBs* are mainly concentrated at both ends of the chromosome. The uneven distribution of the 83 *ZmPUB* genes shows the complex diversity of *U-box* family genes in maize. In addition, chromosomes 2, 5 and 9 have concentrated gene clusters.

The gene duplication events were analyzed to reveal the expansion mechanism of the *ZmPUB* gene family. We found 23 gene pairs involved in gene duplication events. Previous studies defined gene duplication event based on two criteria: alignment sequence length covering ≥ 80% of the longest genes and sequence similarity ≥ 70% within the alignment region [42]. Among these duplicated genes, there were three gene pairs with more than 95% similarity: *ZmPUB16*/*ZmPUB64*, *ZmPUB12*/*ZmPUB30* and *ZmPUB80*/*ZmPUB51*. Furthermore, since *ZmPUB53*/*ZmPUB38* are on the same chromosome and the physical locations of *ZmPUB81*/*ZmPUB18* and *ZmPUB59*/*ZmPUB43* are similar, it can be concluded that *ZmPUB53*/*ZmPUB38* are not crossed on the chromosome.

The gene collinearity results showed a total of 61 tandem duplicate gene pairs on the 10 chromosomes in the maize genome (Fig. 3B and Table S5). Sixty-two collinear *PUB* gene pairs were screened in maize and rice (Fig. 3C and Table S6), while only 3 collinear *PUB* gene pairs were found in *Arabidopsis* and maize (Fig. 3D and Table S7). In *Arabidopsis*, chromosomes 2, 3 and 4 do not show the collinear *U-box* gene. In rice, the collinearity of the *U-box* gene was not shown on chromosomes 7 and 11, but there were different amounts of *U-box* gene collinearity on the other chromosomes.

The rates of non-synonymous (Ka) and synonymous (Ks) substitutions serve as the basis for evaluating the selective pressure in repetitive events. A Ka/Ks ratio of 1 indicates neutral selection, Ka/Ks < 1 suggests purifying selection, and Ka/Ks > 1 implies positive selection. The Ka/Ks ratios for tandem duplications in *ZmPUB* genes were computed using KaKs Calculator 3.0 [43], revealing a range of 0.0547 to 0.6840, with an average of 0.2760 (Fig. S1, Table S8). All Ka/Ks values for duplicated events were less than 1, suggesting that these genes predominantly evolved under purifying selection. Additionally, we calculated the potential dates of duplication events, which occurred between 13.79 and 313.42 million years ago (Mya).

For further exploration of the potential evolutionary processes in the *ZmPUB* gene family, we analyzed the synteny relationships of *ZmPUB* genes in rice, *Arabidopsis* and maize. The Ka/Ks range for *Arabidopsis* and maize was 0.0606 to 0.6422, with an average of 0.2023. In rice and maize, the Ka/Ks range was 0.0281 to 0.8655, with an average of 0.1776.

### Conservative motif and structural analysis of the *U-box* gene family in maize

**Fig. 4:**
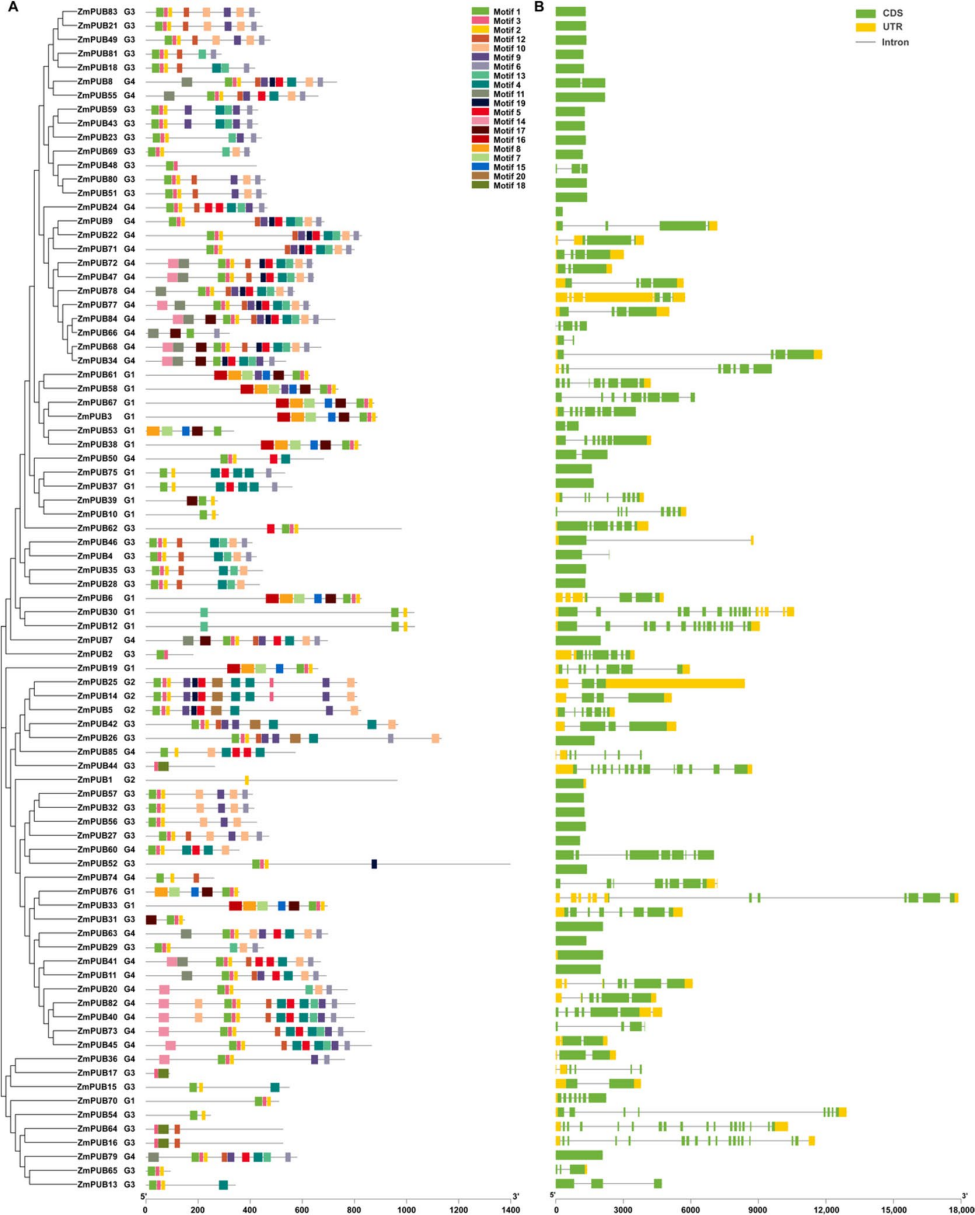
Analysis of the conserved motifs and gene structure of the *U-box* family in maize. **A** The phylogenetics, distribution of conserved motifs. The Neighbor-Joining (NJ) phylogenetic tree of 85 maize *PUB* genes, which were constructed using the MEGA7 software (Neighbor-Joining, NJ) with the bootstrap parameter set to 1000. The subfamily information is represented by G1-G4. Based on MEME online analysis, the 20 conserved motifs of the U-box proteins in maize were designated motifs 1 ~ 20 and are represented by different colors. **B** Distribution of gene structure. The exon/intron structure of *ZmPUB* genes was determined by comparing CDS with genomic sequences. Green and yellow rectangles represents untranslated region (UTR) and coding DNA sequence (CDS) features, respectively, the horizontal line represents gene regions, and gray lines represents introns

All the identified *U-box* genes were analyzed online based on MEME for the presence of novel and undetermined motifs, and 20 conserved motifs of maize U-box proteins were identified (Fig. 4A). U-box proteins exhibit a variable number of motifs, with certain members, like ZmPUB84, possessing up to 14 motifs. Some proteins contain a few motifs, among which the ZmPUB1 protein contains only 1 motif. Further analysis revealed that 80 ZmPUB proteins contained Motif 1 and 45 ZmPUB proteins contained Motif 6. Motif 1 was found in proteins containing Motif 6, suggesting that Motif 1 is related to Motif 6. The majority of U-box proteins contain Motif 1, 2 and 3, with Motif 1 accounting for 94% (80), Motif 2 for 89% (76), and Motif 3 for 83.5% (71). These findings suggest that these three motifs play an important role in the *U-box* gene family. It is also noticeable that Motif 1, 3 and 2 are always adjacent and appear at both ends of the sequence. Motif 5 is always adjacent and appears after Motif 19. Most of the adjacent ZmPUB proteins ( In the same clade within the evolutionary tree and the position is adjacent), including ZmPUB64 and ZmPUB16, ZmPUB59 and ZmPUB43, and ZmPUB83 and ZmPUB21, contain the same motifs. These gene pairs have the same motif type, number and position.

The *ZmPUB* gene structure was analyzed by comparing the CDS and genomic DNA sequences of the *ZmPUB* gene (Fig. 4B). The results showed notable differences in gene structure between the *ZmPUB* genes, with the number of introns ranging from 1 to 17 (Table S9). Within the U-box protein family, thirty-one of the 85 genes in total have no introns, suggesting that the evolution of these genes may follow the transposon mechanism. As shown in Fig. 4B, *ZmPUB83, ZmPUB21, ZmPUB49* and *ZmPUB18* did not contain introns. *ZmPUB64* and *ZmPUB16* contain up to 17 introns. The exon length and arrangement order of the *ZmPUB47* and *ZmPUB77* genes were similar, but the intron length was different.

### Cis-regulatory element analysis in the promoter regions of *U-box* genes

**Fig. 5:**
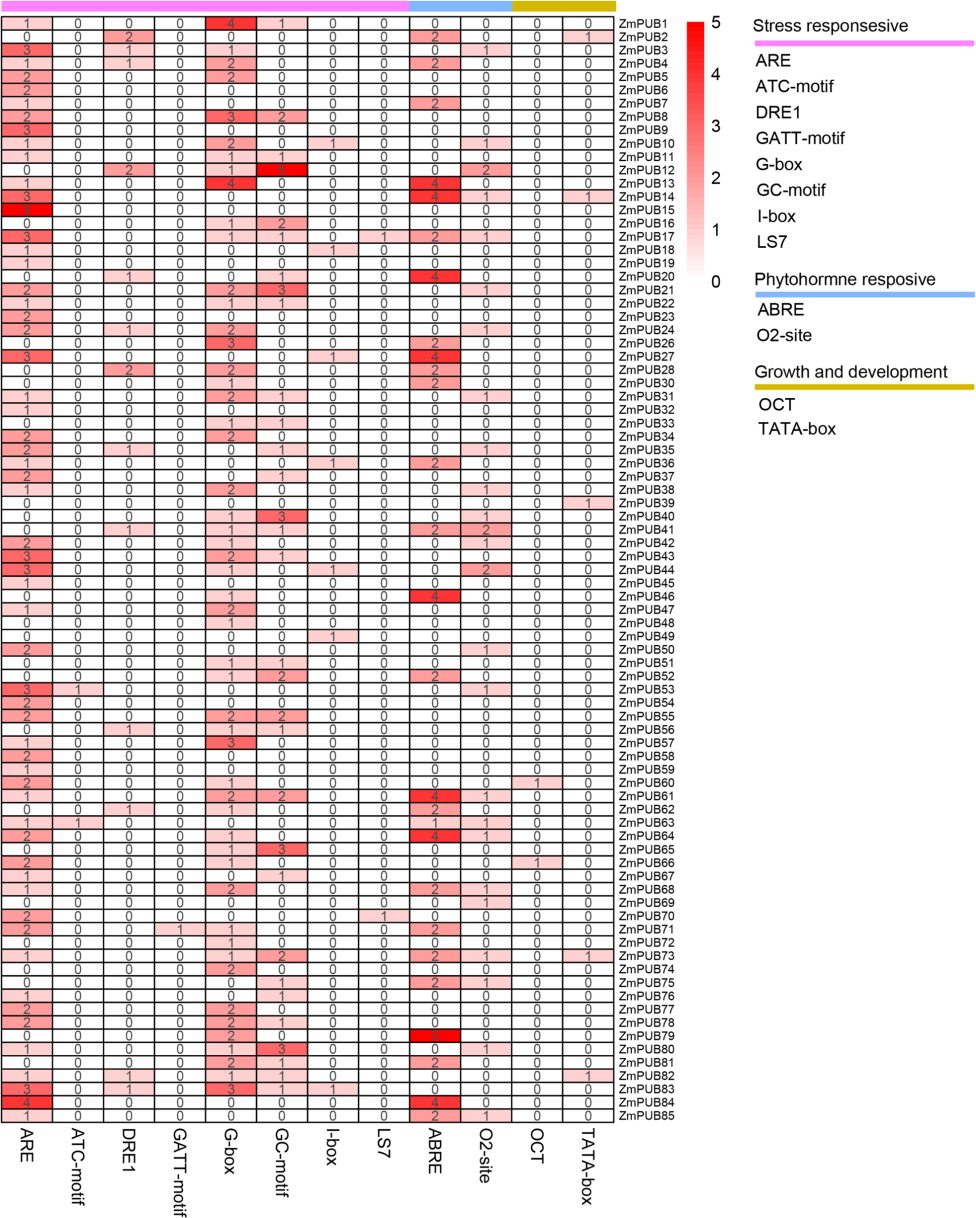
Analysis of the cis-regulatory elements in the promoter regions of *ZmPUB* genes. Different colors represent different cis-regulatory elements located within 1500 bp upstream of *ZmPUBs*, including ARE, ATC motif, DRE1, GATT motif, G-box, GC motif, I-box, LS7, ARBE, O2-site, OTC and TATA-box

The cis-regulatory elements upstream of the start codon are essential for transcriptional regulation of protein-coding genes. *U-box* genes play an important role in the response to various stresses. Cis-acting elements located in the promoter region of genes can regulate the expression of related genes by binding transcription factors. To further investigate the relationship between *ZmPUB* genes and stress, we used the PlantCARE database to predict the potential cis-acting elements of the putative ZmPUBs promoter region (Fig. 5).

An analysis of the 1500 bp upstream region of the *ZmPUB* gene revealed the presence of 12 cis-acting elements. These elements encompass crucial regulatory components, including stress-related elements (ARE, DRE1, ATC-motif, GATT-motif, G-box, GC-motif, I-box, LS7), hormone-related elements (ABRE, O2-site), and defense and pressure-related elements (OCT, TATA-box). It is noteworthy that all *ZmPUB* genes contain cis-acting elements associated with hormone regulation, with a majority linked to stress or hormonal regulation.

We categorized these cis-regulatory elements into three distinct groups based on their involvement in related biological processes: the growth and development process, encompassing two types of elements; phytohormone response, comprising two types of elements; and stress response and includes eight types of elements (Fig. 5 and Table S10). Remarkably, out of the total 373 elements, a substantial majority (71%, 266 elements) belonged to the stress-responsive group. Among these stress-responsive elements, ARE was the most prevalent member, accounting for 39%, while the GATT motif was less frequent, with only one occurrence. In the phytohormone response group, the majority (72%) of the elements are ABREs, implying the likely involvement of *ZmPUB* genes in phytohormone responses. In contrast, the growth and development group exhibited the fewest elements, with only seven in total. Additionally, on average, each *U-box* gene contained four cis-acting elements. *ZmPUB12* and *ZmPUB61* contained the most cis-acting elements, with 10. It seems that the presence of these elements suggested that *ZmPUB* genes could be transcriptionally regulated by abiotic stresses.

### Gene ontology annotation and KEGG pathway enrichment of the maize *U-box* gene

**Fig. 6:**
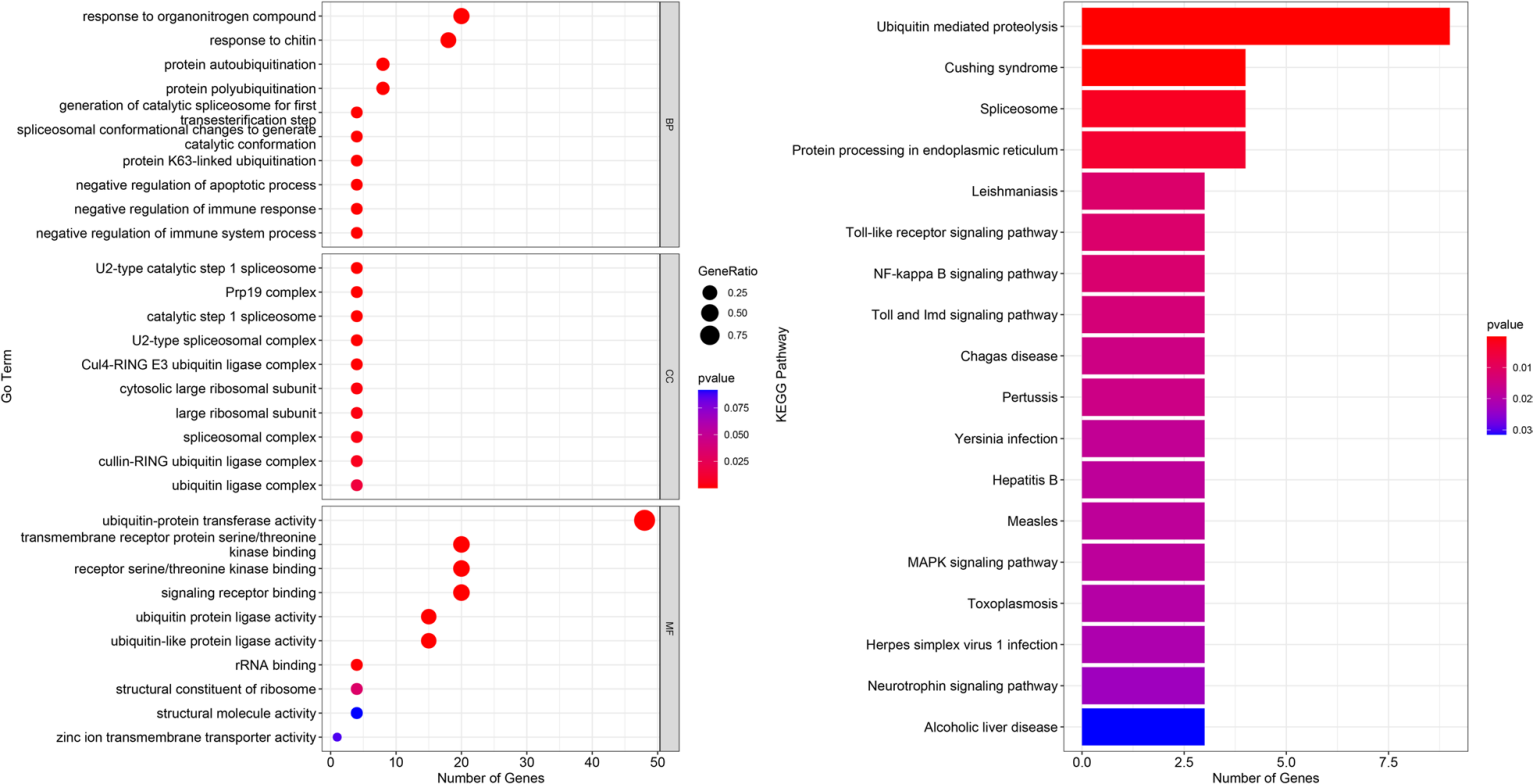
Gene Ontology enrichment and KEGG pathway analysis of the *U-box* gene in maize. **A** GO enrichment shown as dot bubbles. A total of 105 GO terms were notably enriched, covering biological processes, cellular components, and molecular functions. **B** KEGG enrichment shown as a bar graph. The Kyoto Encyclopedia of Genes and Genomes (KEGG) was used to verify the specific metabolic pathways related to these genes

To gain a deeper understanding of the biological function of the *U-box* gene, we conducted GO annotation and KEGG pathway enrichment analysis (Fig. 6). The results show that 105 GO terms covering biological processes, cellular components and molecular functions were notably enriched. Notably, molecular functions were diverse and included key activities such as ubiquitin-protein transferase activity, ubiquitin-conjugating enzyme activity, signal receptor binding and transmembrane receptor protein-serine/threonine kinase binding (Fig. 6A and Table S11). These functions play a crucial role in protein interactions, signal transduction and the regulation of various biological processes in cells. The biological processes involved mainly include the response to organonitrogen compounds, the response to chitin, protein autoubiquitination and protein polyubiquitination. The most prevalent cellular components include the Prp19 complex, the U2 spliceosome complex and the Cul4-RING E3 ubiquitin ligase complex.

We performed a comprehensive analysis of the *U-box* gene to investigate its functionality in metabolic and signal transduction pathways using eggnog-mapper. The results show that the *U-box* gene contributes notably to 21 pathways, including ubiquitin-mediated proteolysis, Cushing syndrome, spliceosome and protein processing in the endoplasmic reticulum (Fig. 6B and Table S12). In particular, the ubiquitin-mediated proteolysis pathway was the most represented, with nine *U-box* genes.

### Expression analysis of the U-box protein-encoding genes in different tissues

**Fig. 7:**
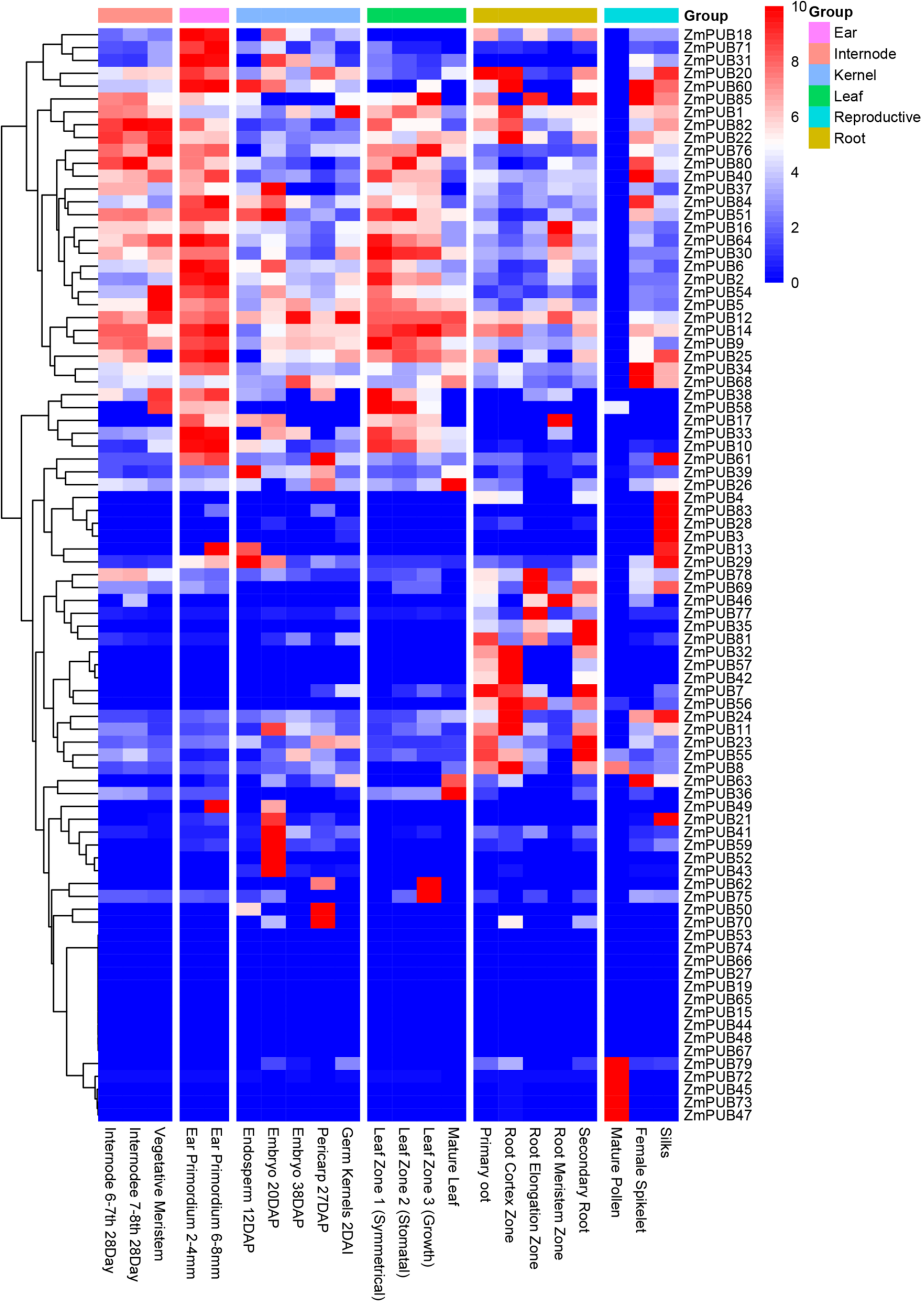
Expression of the U-box protein family genes in each tissue. Different colors at the top represent different groups (Ear, Internode, Kernel, Leaf, Root and Reproductive), and each of the following columns represents 22 tissues. The standardized FPKM values are shown on the top right scale, with red representing high expression and blue representing low expression

To explore the function of *U-box* genes in different tissues, we were able to examine the expression of *ZmPUB* genes in 22 tissues using an existing database (https://www.maizegdb.org/expression). These tissues included the ear, internode, kernel, leaf, reproductive tissue and root (Fig. 7 and Table S13). Some *ZmPUB* genes were expressed only in certain tissues. For example, *ZmPUB45, ZmPUB47, ZmPUB72* and *ZmPUB73* were expressed in mature pollen; *ZmPUB32, ZmPUB35, ZmPUB57* and *ZmPUB42* were mainly expressed in roots; *ZmPUB41*, *ZmPUB43* and *ZmPUB53* were mainly expressed in kernels; and *ZmPUB21* and *ZmPUB59* were mainly expressed in kernels and reproductive tissue. In the ear group, *ZmPUB6, ZmPUB10, ZmPUB13, ZmPUB18, ZmPUB25, ZmPUB31*, *ZmPUB33, ZmPUB49, ZmPUB64, ZmPUB71* and *ZmPUB84* had high expression levels, whereas *ZmPUB13* and *ZmPUB49* were expressed only in the longer ear primordium. Some *ZmPUB* genes, such as *ZmPUB15, ZmPUB19, ZmPUB27, ZmPUB44, ZmPUB48, ZmPUB53, ZmPUB65, ZmPUB66, ZmPUB67* and *ZmPUB74,* were barely expressed in the 22 tissues and may have more specific functions. Because the *ZmPUB* gene plays a crucial role in the response to stress, plant growth and development, expression pattern analysis can provide important clues for the study of function.

### Expression pattern of *ZmPUB* genes under abiotic stress

**Fig. 8:**
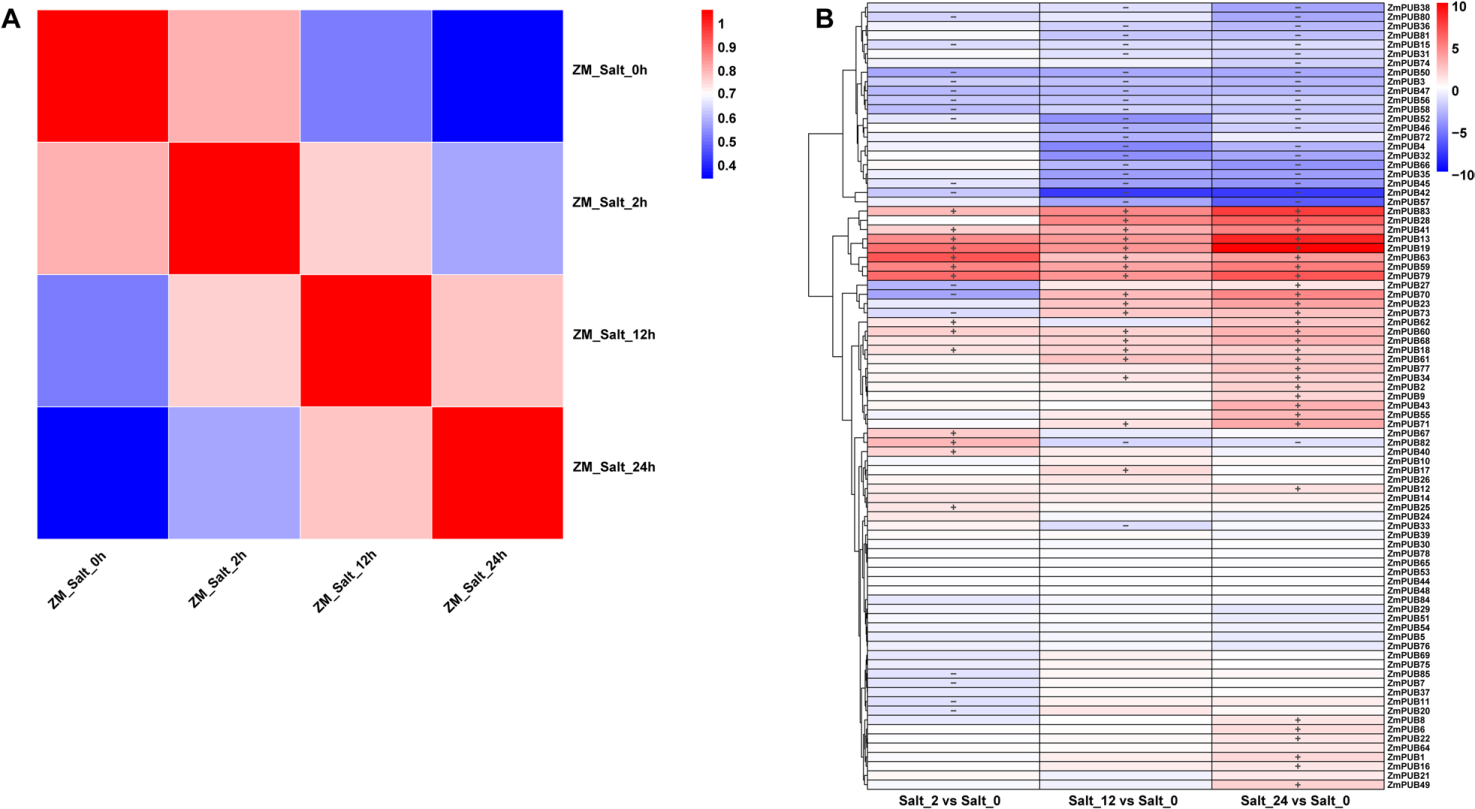
Correlation analysis of the salt stress samples across time periods and expression heatmap of the *ZmPUB* genes. **A** Correlation analysis between samples exposed to 0, 2, 12 and 24 h of salt stress treatments. Color intensity reflects the strength of the correlation, with darker colors representing stronger correlations. **B** Expression heatmap of *ZmPUB* genes under 0, 2, 12 and 24 h of salt stress treatment. The x-axis denotes different samples of salt stress, while the y-axis represents *ZmPUB* genes ordered based on hierarchical cluster analysis. The color scale in the upper right corner corresponds to the standardized log2 fold changes (log2FC) values. Genes were considered upregulated if their expression increased at 2, 12, and 24 h with a log2 fold change > 1 for all three time points, and downregulated if their expression decreased at these time points with a log2 fold change < -1. “ + ” representing up-regulated and “-” representing down-regulated

We conducted gene expression analysis of *U-box* family genes in maize under salt stress at 2, 12 and 24 h with 0-h treatment as a control (Fig. S2). Genes were considered to have differences in expression levels if |log_2_N|≧1. The correlation analysis of all transcriptome samples showed a difference between the control and treatment groups, and the differences deepened significantly with treatment time (Fig. 8A). After salt treatment, *ZmPUB13*, *ZmPUB18*, *ZmPUB19*, *ZmPUB41*, *ZmPUB59*, *ZmPUB60*, *ZmPUB63*, *ZmPUB68*, *ZmPUB79* and *ZmPUB83* were upregulated (considered upregulated if their expression increased at 2, 12 and 24 h with a log2 fold change > 1 for all three time points). *ZmPUB3, ZmPUB42, ZmPUB47, ZmPUB50, ZmPUB56* and *ZmPUB58* were downregulated (considered downregulated if their expression decreased at 2, 12 and 24 h with a log2 fold change < -1 for all three time points) (Fig. 8B and Table S14).

**Fig. 9:**
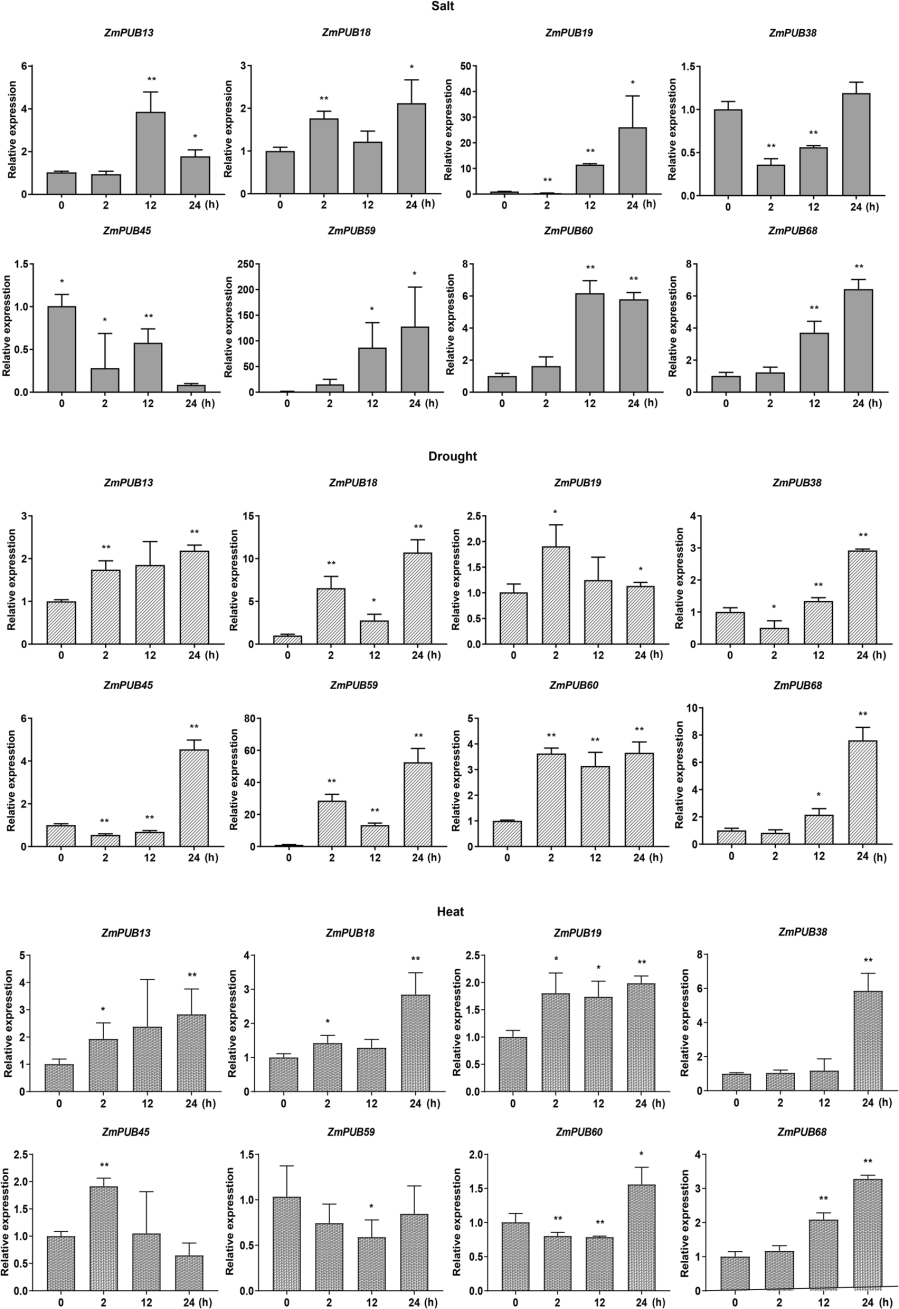
qRT-PCR analysis of the *U-box* gene under salt, drought and high temperature stress. The 4-week-old seedlings were subjected to salt, drought and high temperature stress for 0, 2, 12 and 24 h. Eight abiotic stress-responsive genes were selected for qRT-PCR experiments, and the results were consistent with the RNA-seq data: *ZmPUB13*, *ZmPUB18*, *ZmPUB19*, *ZmPUB59*, *ZmPUB60* and *ZmPUB68* were upregulated, while *ZmPUB38* and *ZmPUB45* were notably downregulated. Some *U-box* genes were induced by more than one stress condition. For example, *ZmPUB13, ZmPUB18, ZmPUB19* and *ZmPUB68* were induced by salt, drought and heat treatments

To verify the high-throughput sequencing data, 8 genes with relatively high up-regulation or down-regulation responding to salt stress were selected for qRT-PCR experiments and were consistent with the transcriptome analysis (Fig. 9). Under salt stress, *ZmPUB13, ZmPUB18, ZmPUB19, ZmPUB59, ZmPUB60* and *ZmPUB68* were upregulated, while *ZmPUB38* and *ZmPUB45* were notably downregulated. Among them, the transcription levels of *ZmPUB19* and *ZmPUB59* increased notably, and the upregulation rates reached tenfold and 80-fold, respectively, after 12 h of salt stress treatment. After 24 h of salt stress treatment, *ZmPUB59* was upregulated 120-fold. Considering that the *U-box* genes were induced by more than one stress condition, we conducted qRT-PCR to examine the 8 genes under drought and heat stress conditions. Under drought stress, *ZmPUB13, ZmPUB18, ZmPUB19, ZmPUB59, ZmPUB60* and *ZmPUB68* were notably upregulated, while *ZmPUB38* and *ZmPUB45* were notably downregulated in the early stage ( 2h and 12h) of treatment (Fig. 9). Under heat stress, *ZmPUB13, ZmPUB18, ZmPUB19, ZmPUB38* and *ZmPUB68* were notably upregulated, while *ZmPUB59* and *ZmPUB60* were notably downregulated after 12 h of treatment. The results showed that *ZmPUB13, ZmPUB18, ZmPUB19* and *ZmPUB68* were induced by salt, drought and heat treatments. Furthermore, *ZmPUB38* and *ZmPUB45* were downregulated during the early stages of salt and drought treatments, but they were upregulated under heat stress.

### Subcellular localization of *ZmPUB19* and *ZmPUB59*

**Fig. 10:**
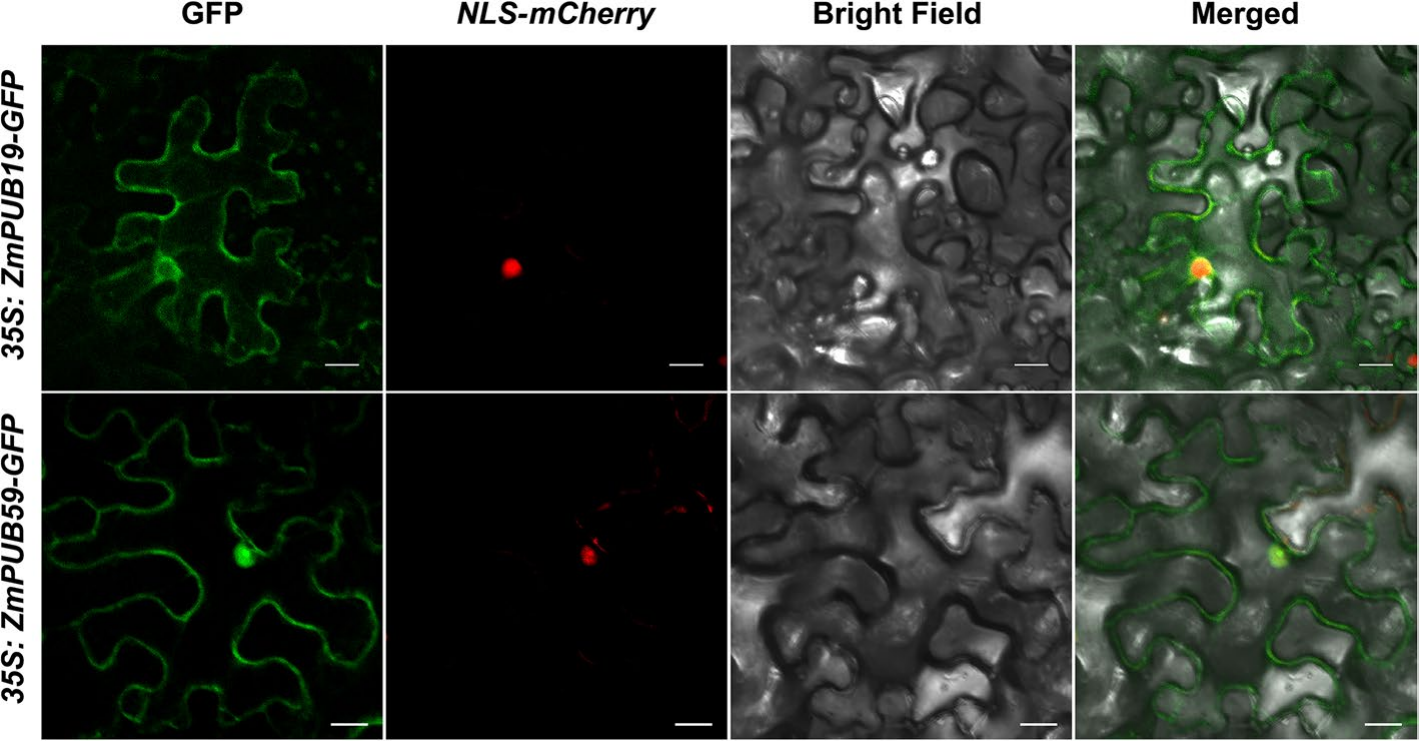
Analysis of the subcellular localization of *ZmPUB19* and *ZmPUB59*. *ZmPUB19*-GFP and *ZmPUB59*-GFP colocalized with NLS-mCherry. Paired constructs were infected into Nicotiana leaves. GFP and mCherry fluorescence signals were monitored 40–48 h after infection by confocal microscopy. For each construct, 10–20 cells were analyzed, and a similar nuclear localization pattern was observed. *ZmPUB19*-GFP and *ZmPUB59*-GFP had strong fluorescence expression signals in the nucleus and coincided with the NLS-mcherry expression signal in the nucleus, confirming that *ZmPUB19* and *ZmPUB59* were mainly localized in the nucleus. Scale bar, 20 μm

To further elucidate the biological function of the *ZmPUB* gene in maize under abiotic stress, we selected the two genes, *ZmPUB19* and *ZmPUB59*, which exhibited the highest upregulation under salt stress conditions according to the results of qRT-PCR and RNA-seq, for subcellular localization analysis. The *35S:ZmPUB19-GFP* or *35S:ZmPUB59*-GFP fusion expression vector and the nuclear marker protein (*NLS-mcherry*) were coexpressed in tobacco leaves (*Nicotiana benthamiana*) by *Agrobacterium*-mediated osmosis. The results showed strong fluorescence expression signals of *35S:ZmPUB19-GFP* and *35S:ZmPUB59-GFP* and were consistent with the expression signals of the nuclear marker protein (*NLS-mcherry*) (Fig. 10), thus confirming that *ZmPUB19* and *ZmPUB59* are mainly localized in the nucleus.

## Discussion

Abiotic stresses such as salinity, drought and high temperature, profoundly impact maize growth [44]. The ubiquitin pathway emerges as a critical player in posttranslational protein modifications, with U-box proteins, functioning as E3 ligases, play essential roles in plant growth and stress responses. Despite that several studies have focused on individual maize *U-box* genes and RNA-seq has been conducted to assess the impact of hormones (ABA, IAA, and GA) on the expression of ZmPUB [45], a comprehensive genome-level analysis of the maize U-box protein family's response to abiotic stress has been lacking.

In this study, we identified a total of 85 *ZmPUB* genes in maize (Table 1), classified into four subfamilies through phylogenetic analysis (Fig. 1). Additional functional domains were discovered in 85 U-box protein families (Fig. 2). These domains are essential for plant defense and disease resistance. Previous studies have shown that the ARM domain is the second most conserved domain in rice and *Arabidopsis* and is associated with substrate recognition in the ubiquitination process. In maize, 21 ZmPUB proteins have the ARM domain and is similar to those in rice and *Arabidopsis.* ARM repeats are characteristic repetitive amino acid sequences identified in proteins, with a length of approximately 40 residues, and proteins containing these sequences typically have multiple tandem copies [46, 47]. In general, E3 ubiquitin ligases have protein–protein interaction domains that interact with their substrates to facilitate ubiquitination [48]. For instance, the ARM repeat protein *AtPUB-ARM* in *Arabidopsis* regulates various cellular processes [49]. *PUB-ARM* genes have been reported to respond to diverse abiotic stresses in different plant species. Cho et al. found that the *PUB-ARM* gene was affected by salinity stress in hot pepper [50]. In *Arabidopsis, t*wo *PUB-ARM* genes, *AtPUB18 and 19,* were found to be transiently expressed under ABA treatment [51]. In cabbage, the homologous *BrPUB-ARM* genes *BrPUB6 and BrPUB54* were upregulated under ABA treatment. These findings emphasize the important role of *PUB* genes in response to various environmental pressures.

Gene duplication (GD), mutation and natural selection are the main sources of new genes with novel functions and provide the foundation for biological diversity [52]. Chromosomal analysis revealed random distribution of the 85 *ZmPUB* genes across maize chromosomes, with tandem duplicates and collinearity observed. The association between maize *PUB* genes and rice *PUB* genes is stronger compared to *Arabidopsis* and is consistent with the results of homologous evolution analysis between maize and rice (Fig. 3). It is possible that homologous *PUB* genes perform similar functions in maize and rice. Tandem repeats, as a major component of *U-box* gene extensions, can effectively contribute to maintaining gene structure and function. This may also be a mechanism for acquiring new functional domains in genes [53]. The whole-genome duplication (WGD) has significant implications for species evolution, as it generates new gene copies, contributing significantly to adaptability, isolation, phenotypic robustness, and evolution. These events result in the emergence of numerous complex gene families in descendant species [54]. In addition to multigene duplications, there are also numerous single-gene duplications. Although these single-gene duplications often result in gene loss [55], the retained genes have important implications for evolution [56]. The analysis of the Ka/Ks ratios for tandem duplications in *ZmPUB* genes revealed values predominantly less than 1, indicating that these genes primarily evolved under purifying selection. Notably, the comparison of the Ka/Ks ratios between *Arabidopsis* and maize, and rice and maize, showed lower values for the latter pairs. This suggests that maize *U-box* genes may have experienced stronger purifying selection as compared to their counterparts in *Arabidopsis* and rice. The lower Ka/Ks values for rice and maize may reflect a more stringent elimination of deleterious mutations, potentially contributing to the conservation and functional stability of the *U-box* gene family in these species. This suggests that the *U-box* gene family in maize has undergone relatively stronger selective pressure, possibly contributing to the high functional conservation of these genes. Consequently, these findings point to the potential impact of selective pressures on the evolutionary processes and functional characteristics of the *U-box* gene family in maize.

In eukaryotic organisms, genes typically consist of exons, introns and 5' and 3' untranslated regions (UTRs). Previous studies have shown that gene structure is closely linked to functions and evolution [57]. In the maize *PUB* gene family, there is a clear difference in the number of exons (Fig. 4). Further analysis revealed that all proteins containing Motif 6 also had Motif 1, suggesting an association between Motif 1 and Motif 6. The majority of U-box proteins contain Motif 1, 2, and 3, with approximately 94% (80) containing Motif 1, 89% (76) containing Motif 2, and 83.5% (71) containing Motif 3, suggesting that these three motifs play an important role in the *U-box* gene family. The ZmPUB proteins had similar motifs in the common subfamily, while they mostly varied among different subfamilies. It is possible that there is functional redundancy within the same subfamily [58]. Analysis of gene structure revealed that there was considerable variation in gene structure among *ZmPUB* genes (Table S9). Many *ZmPUB* genes have similar exon lengths, permutation orders and different intron lengths, which suggests that these genes may have undergone a degree of conservation during the process of evolution. Intron lengths can impact gene expression, alternative splicing, and regulatory processes. The similarity in exon lengths and arrangement suggests shared requirements for encoding core protein information, while the variation in intron lengths may signify regulatory differences and contribute to functional diversity.

Analyzing the gene structure provides further insights, revealing variations in the number of exons and introns among *ZmPUB* genes. In this study, the number of exons and introns varied in each subfamily (Fig. 4). Analysis of the number of exons and introns can provide more information about the evolution of exon–intron structures and the underlying mechanisms behind different biochemical functions. *ZmPUB* genes from the same group exhibited similar gene structures, implying functional conservation within the maize *PUB* gene family. Additionally, variations in exon numbers among *ZmPUB* genes suggest functional diversity within the maize *PUB* gene family. The conservation analysis of the gene motifs showed that the *PUB* genes retained the U-box domain and accumulated additional motifs over the course of evolution. The presence of similar motifs among *ZmPUB* genes suggests conserved evolutionary relationships and similar biological functions. Exons carry core information required for protein synthesis, while introns protect coding proteins from random harmful mutations [59]. In the *PUB* gene family, several intronless genes have been reported in multiple species, such as cabbage [24], tomato [25] and *Medicago truncatula* [60], suggesting the strong structural integrity of *PUB* genes. These analyses are of great value to scientists interested in the evolution, structure and function of the *PUB* gene family. Further analysis of the number of exons and introns can provide more information about the evolution of exon–intron structures and the underlying mechanisms behind different biochemical functions.

Exploring the cis-acting elements in *ZmPUB* genes contributes to revealing their roles in stress response and hormone regulation. We identified 12 major cis-acting elements in the *ZmPUB* genes (Fig. 5). This suggests that the maize *U-box* gene family is actively involved in plant development and responses under stress conditions and that the expression of the *ZmPUB* gene may be stress-regulated and hormonally induced and regulated. In the phytohormone response group, 72% of elements were ABREs, which made up the largest number of elements. ABRE can transcribe and regulate abscisic acid response genes [61], while ABRE_ERD1 has been shown to be upregulated in response to both water stress and etiolation in *Arabidopsis*, suggesting the essential role of ABRE_ERD1 in dehydration induction and stress response [62]. The *PUB* gene contains diverse types and quantities of cis-acting elements, suggesting the specificity of the maize *PUB* gene family in various biological processes. It seems that the presence of these elements implied that *ZmPUB* genes could be transcriptionally regulated by abiotic stresses.

In our research, the GO annotation and KEGG pathway enrichment analysis provided valuable insights into the diverse roles of ZmPUB proteins, uncovering their involvement in a wide range of biological processes, cellular components, and molecular functions (Fig. 6A). Our findings unveiled the association of ZmPUB proteins with responses to chitin and organonitrogen compounds, indicating their potential role in orchestrating responses to environmental stimuli. Moreover, the results of the KEGG pathway analysis emphasized the significant contribution of *U-box* genes to various pathways, including those associated with essential cellular processes such as ubiquitin-mediated proteolysis, protein processing in the endoplasmic reticulum, the spliceosome, as well as pathways related to conditions such as Cushing syndrome (Fig. 6B). The significant representation of *U-box* genes in the ubiquitin-mediated proteolysis pathway further underscores their pivotal role in regulating protein degradation and maintaining cellular homeostasis. The outcomes of the GO annotation and KEGG pathway enrichment analyses portray the maize *U-box* gene family as multifaceted and pivotal in various biological processes, stressing its involvement in stress responses, hormone regulation, and other cellular processes. These insights align with the comprehensive functional significance exhibited by the diverse cis-acting elements within *ZmPUB* genes, underlining their potential regulatory role in stress responses and hormone regulation, and reaffirming the coordination between GO and KEGG analyses with the cis-acting elements' analysis.

The tissue expression profiles of *ZmPUB* genes provide a nuanced understanding of their roles during different developmental processes. Here, we identified expression profiles of maize *PUB* genes during different tissue development processes (Table S13). The tissue expression heatmaps showed that some *ZmPUB* genes were specifically expressed in certain tissues (Fig. 7). Some *ZmPUB* genes were barely expressed in 22 tissues, such as *ZmPUB15, ZmPUB19, ZmPUB27, ZmPUB44, ZmPUB48, ZmPUB53, ZmPUB65, ZmPUB66, ZmPUB67* and *ZmPUB74* and may have more specific functions. Gene expression patterns are frequently linked to their functions, and analyzing expression patterns can provide vital insights for studying function.

Studies have shown that *U-box* genes play a crucial role in tolerance to various abiotic stresses, such as salt, drought and heat. Using high-throughput sequencing data, we analyzed the gene expression levels 2, 12 and 24 h after high-salt stress treatment and found different expression of 16 *ZmPUB* genes under nonbiological stress treatments (Fig. 8B). The qRT-PCR results for genes responding to abiotic stress were consistent with the transcriptome analysis (Fig. 9). In general, more genes were upregulated than downregulated under drought, salt and cold stress. Among the upregulated genes, *ZmPUB13, ZmPUB18, ZmPUB19* and *ZmPUB68* were consistently upregulated under all three abiotic stress conditions. In particular, these genes were more strongly correlated between salt and drought stress. The expression of *ZmPUB59* was upregulated under drought and salt stress but downregulated under high-temperature stress. Interestingly, we observed notable differences in expression at the three time points during the salt stress response, implying different roles for these genes under salt stress. *ZmPUB13* and *ZmPUB60* were strongly expressed at 12 h under salt stress but at 24 h under drought and high-temperature stress, suggesting that *ZmPUB13* and *ZmPUB60* respond faster to salt stress than to drought and high-temperature stress. These results show that maize responds differently to abiotic stress at different time points and under different treatments. Several *U-box* genes were induced under all three stress conditions, suggesting their important role in abiotic stress responses. Further studies are needed to elucidate the specific functions of these *ZmPUB* genes.

Through homologous analysis between maize and other species, potential similarities in their responses to various abiotic stresses can be revealed. In rice, *ZmPUB80* and *OsPUB75* are characterized homologous genes. *OsPUB75* encodes a cytoplasmic E3 ubiquitin ligase of the RING type and serves as a crucial negative regulator in response to abiotic stress. Specifically, under salt stress, the transcription of *OsPUB75* is inhibited [63]. Notably, *ZmPUB80* in maize exhibited a similar expression pattern to *OsPUB75,* showing notably downregulated under high-salt stress. This suggests the potential involvement of *ZmPUB80* in the negative regulation of the abiotic stress response. Furthermore, *ZmPUB21* and *OsPUB41* showed 100% homology (Fig. 1). *ZmPUB21* was notably upregulated under salt stress, akin to the role of *OsPUB41* as a key negative regulator in the regulation of drought stress tolerance [11]. Similarly, *ZmPUB22* and *OsPUB15* are homologous genes. *OsPUB15* is a negative regulator of cell death and plant response to abiotic stress, conferring high tolerance to salt [10]. Additionally, *ZmPUB7, 11, 41* and *OsPUB2, 3* are homologous genes, and the homologous U-box E3 ubiquitin ligases *OsPUB2* and *OsPUB3* are involved in the positive regulation of the response to low-temperature stress [64]. Studies have shown that *OsPUB3* acts as a positive regulator of cold stress, and compared to that in wild-type plants, its overexpression in transgenic plants enhances cold stress tolerance [64]. *ZmPUB41* was upregulated after 24 h of salt stress treatment. Given its high homology with *OsPUB2/3*, it is inferred that *ZmPUB41* may be involved in the positive regulation of abiotic stress. Moreover, *OsPUB45* is homologous to *ZmPUB59*. *OsPUB45* is notably upregulated under drought, salt and cold stress in rice [65], mirrors the expression pattern of *ZmPUB59*, which exhibited a 4.8-fold upregulation after 2 h of salt stress treatment. The similar expression patterns of Z*mPUB59* and *OsPUB45* suggest that these two genes may have similar functions. In *Arabidopsis*, *AtPUB18* and *AtPUB19* are positively regulated by ABA and salt, and compared to wild-type plants, their double mutants were less sensitive to ABA and salt inhibition of germination [66]. Notably, under salt stress treatment, *ZmPUB79* was upregulated by 6.61-fold and showed homology with *AtPUB18* and *AtPUB19*, suggesting that it may be involved in the positive regulation of the salt stress response. A comprehensive analysis of the maize response to abiotic stress is beneficial for selecting specific *U-box* genes for functional research, enhancing maize stress resistance and breeding superior maize varieties and is important for increasing maize yields.

Our investigation into the subcellular localization of *ZmPUB19/59* proteins in the nucleus adds a spatial dimension to their biological functions. The subcellular localization experiment showed that *ZmPUB19/59* proteins are located in the nucleus, suggesting that *ZmPUB19/59* may exert biological functions in the nucleus (Fig. 10). The high expression levels of *ZmPUB19* and *ZmPUB59* suggest that they play a crucial role in the abiotic stress response, with the upregulation level of *ZmPUB19* reaching tenfold and *ZmPUB59* reaching 80-fold after 12 h of salt stress treatment. After 24 h of salt stress treatment, *ZmPUB59* was upregulated 120-fold. *ZmPUB59* is homologous to *OsPUB45*. In rice, *OsPUB45* is considered a positive regulator of plant defense and stress signaling responses [67], suggesting the important role of *ZmPUB59* in the response to abiotic stress. However, the cellular mechanisms of *ZmPUB59* in the response to abiotic stress are still unclear and need to be further explored in future studies.

## Conclusions

We conducted a systematic bioinformatic analysis of the maize *U-box* gene family, including analyses of gene structure, conserved motifs, chromosome localization, cis-acting elements, phylogenetic evolution, gene expression and subcellular localization. We predicted the key role of the *U-box* gene family in maize plant development and response to stress and identified key genes associated with abiotic stress. We analyzed the gene expression of maize under salt, drought and high-temperature stresses, revealing tissue-specific patterns for *U-box* genes. The conserved *U-box* genes likely play a critical role in maize adaptation to stress, and multiple *U-box* genes may cooperate to protect plants from abiotic stress. Our results provide a basis for further analysis of *ZmPUB* genes to determine their function and elucidate the molecular mechanisms underlying the response of maize to abiotic stress.

## Methods

### Identification of *ZmPUB* gene family members in maize

The whole-genome sequences and protein sequences of B73 maize were obtained from MaizeGDB (https://curation.maizegdb.org/) and the Phytozome database (https://phytozome-next.jgi.doe.gov/). The HMM information of the U-box domain (PF04564) was obtained from the Pfam database (https://www.ebi.ac.uk/interpro/entry/pfam/), and the candidate protein sequence containing the U-box domain was selected using HMMER 3.0 (http://hmmer.janelia.org/). The domain information of the candidate protein was analyzed to identify proteins containing U-box domains as ZmPUB proteins in maize using Pfam with an E-value cutoff level of 1.0. The domain was visualized based on the ZmPUB family protein domain from the Pfam database. The UniProt database (https://www.uniprot.org) was used to locate information related to the ZmPUB proteins identified, including domain type, number of amino acids, isoelectric points, relative molecular weight, chromosome position and more.

### Analysis of chromosome localization, gene structure, collinearity and cis-acting elements in the promoter region of maize

The chromosomal positions and gene structure of the *U-box* genes were obtained from the MaizeGDB and Phytozome databases. The similarity of each gene sequence was compared using ClustalOmega (https://www.ebi.ac.uk/Tools/msa/clustalo/). The chromosome location mapping was completed by MapChart software [68]. To assess gene duplication, a BLAST sequence comparison of the *U-box* genes in maize, *Arabidopsis* and rice was performed using an e-value < 1e^−10^ (lower confidence is higher) as a cutoff value. The exon/intron structure of the *ZmPUB* genes was determined by comparing CDS with genomic sequences using TBtools software for visualization [69]. MCScanX was used to assess repeated events and analyze collinear associations between species (parameters: match score: 50; match size: 5; gap penalty: -1; overlap window: 5; E-value threshold: 10^–5^; max gaps: 25) [70]. The 1500 bp DNA upstream sequence of the transcription start site of the *U-box* family gene sequence was downloaded from MaizeGDB. The cis-acting elements were analyzed using the PlantCARE database (http://bioinformatics.psb.ugent.be/webtools/plantcare/html/) and TBtools software was used to display [69].

### Analysis of motif, phylogenesis and tissue-specific expression of *ZmPUB* gene family members

The U-box protein sequences of maize, rice and *Arabidopsis* were obtained from the UniProt database. The conserved motifs of ZmPUBs were obtained from MEME (https://meme-suite.org/meme/). The MEME results were displayed with TBtools software [69]. The sequences of the U-box proteins of the three plants were aligned by ClustalX (http://www.clustal.org/), and then the phylogenetic trees of maize, rice and *Arabidopsis* were constructed by MEGA7 software (Neighbor-Joining, NJ) with the bootstrap parameter set to 1000. RNA-seq data on maize *U-box* gene expression in different tissues were obtained from MzizeGDB. The 22 tissues spanning the vegetative and reproductive stages of maize development were analyzed to generate a comprehensive and integrated gene expression profile [71]. The results were displayed using R software, where *ZmPUB* genes were ordered based on a hierarchical clustering analysis.

### Gene Ontology (GO) annotation and KEGG pathway enrichment of the maize *U-box* gene

Proteomic data for maize were obtained from the MaizeGDB database. Functional annotation was performed using eggnog-mapper (http://eggnog-mapper.embl.de) [72]. To better understand the functions of *U-box* genes, we performed analysis of GO terms and KEGG pathways with the R package clusterProfiler and plotted them by using the ggplot2 package in R [73–75]. *P* values < 0.05 were considered statically significant [76].

### Plant materials and stress treatments

Seeds of maize (*Zea mays* L. inbred strain B73) were sterilized with 3% NaClO for 20 min and washed three times in sterilized water. Then, the seeds were incubated in saturated CaSO_4_ solution for 6 h. After soaking, the seeds were inoculated onto sterilized filter paper and were kept moist at 26 °C to germinate in the dark for 48 h. Seedlings at the one-leaf stage were transplanted into point-hole plastic plates under the following conditions: 28℃, 1/2-strength Hoagland nutrient solution and a photoperiod of 12/12 h (day/night). The nutrient solution was returned to normal strength after two days. Seedlings at the four-leaf stage were subjected to three abiotic stress treatments. Salt treatment was initiated by nutrient solution containing 300 mM NaCl. Drought treatment was initiated by moving the plants to dry filter paper. High-temperature treatment was initiated at 38℃. After treatment for 0, 2, 12 and 24 h, the seedlings were harvested and stored in liquid nitrogen and then stored in the refrigerator at -80 ℃ for subsequent experiments. The total RNA samples were sent to Genergy Biotechnology (Shanghai) for sequencing on an Illumina® Hiseq3000. Each treated sample consisted of three seedlings. Subsequently, the raw data were processed to eliminate adapter sequences and low-quality reads using Skewer software (v0.2.2) [77]. The resulting clean reads were aligned to the reference genome sequence of maize using STAR software (v2.5.3a) [78].

### Quantitative RT-PCR analysis

Total RNA from the stored plants was extracted with TRIzol, and cDNA was obtained using the BeyoRT™ II cDNA kit (Beyotime, Shanghai, China). Quantitative analysis was performed by real-time PCR in conjunction with Hieff® SYBR Green Master Mix (Yeasen) on a LightCycler 480 System (Roche, Vienna, Austria) according to the manufacturer’s instructions. The primers used are listed in Table S15. The following reaction conditions were applied: predenaturation at 95 °C for 30 s, followed by 40 cycles of denaturation at 95 °C for 5 s and 65 °C for 60 s, and the melting curve was evaluated from 65 °C to 95 °C. The relative transcript levels of the candidate genes were calculated according to the 2^−ΔΔCT^ method. The melting peaks of the candidate genes are displayed in Fig. S3.

### Construction of vectors and subcellular localization

The subcellular localization of the *U-box* family genes was predicted using Plant-PLoc (http://www.csbio.sjtu.edu.cn/bioinf/plant) by uploading protein sequences. Two *U-box* genes, *ZmPUB19* and *ZmPUB59,* were cloned using In-Fusion technology. Primers were designed using online software, and sequence information can be found in Additional file 4: Table S15. The PCR products of *ZmPUB19* and *ZmPUB59* were linked to the 5’ end of the mGFP of the pGreenII-35S-mGFP cloning vector and then transformed into DH5α*.* The resulting fusion expression vectors, pGreenII-*ZmPUB19* and pGreenII-*ZmPUB59*, were validated by sequencing. Subsequently, the validated fusion expression vectors were transformed into *Agrobacterium* strain GV3101 (pSoup). The nucleus marker was created by linking the NLS sequence to pGreenII-35S-mCherry and was tested with infected *Nicotiana* leaf cells [79]. The constructed vector plasmid was transformed into *Agrobacterium* strain GV3101 (pSoup), and the vectors were transiently transformed into *Nicotiana* leaves [80]. The infected tobacco plants were cultured in the dark at 25 °C for 12 h and then returned to normal culture conditions for 24 h. Finally, the infected cells of the lower epidermis of the transformed leaves were imaged using a confocal laser microscope (OLYMPUS FV3000, Japan) at an excitation wavelength of 488 nm to detect GFP and at an excitation wavelength of 561 nm to detect mCherry.

### Statistical analysis

In this study, the qRT-PCR results are reported as the means of three independent experiments. The significant differences between treatments were analyzed using standard deviation and one-way analysis of variance (ANOVA). The significant differences between the control group and the treatment groups were assessed by Student’s t test. The result of *P* < 0.05 was used as the significance threshold (* *P* < 0.05, ** *P* < 0.01 and *** *P* < 0.001).

### Supplementary Information

**Additional file 1.**

**Additional file 2.**

**Additional file 3.**

**Additional file 4.**

## Data Availability

The datasets generated during the current study are available in the GenBank repository (http://www.ncbi.nlm.nih.gov/Genbank) and the MaizeGDB (http://www.maizegdb.org), and their public access to these databases is open. All data generated or analyzed during this study are included in this article and its supplementary information files.

## Abbreviations

E3: E3 ubiquitin ligase enzyme
NJ: Neighbor-joining
PUB: Plant U-box
ZmPUB: Maize PUB
