# Supplementary material for: Genome-wide identification and transcriptome profiling expression analysis of the U-box E3 ubiquitin ligase gene family related to abiotic stress in maize (Zea mays L.)
Source: BMC Genomics. 2024 Feb 1;25:132. doi: 10.1186/s12864-024-10040-8 (PMC10832145; doi:10.1186/s12864-024-10040-8)
Supplement: Supplementary file 1 — Additional file 1. [file 12864_2024_10040_MOESM1_ESM.pdf]

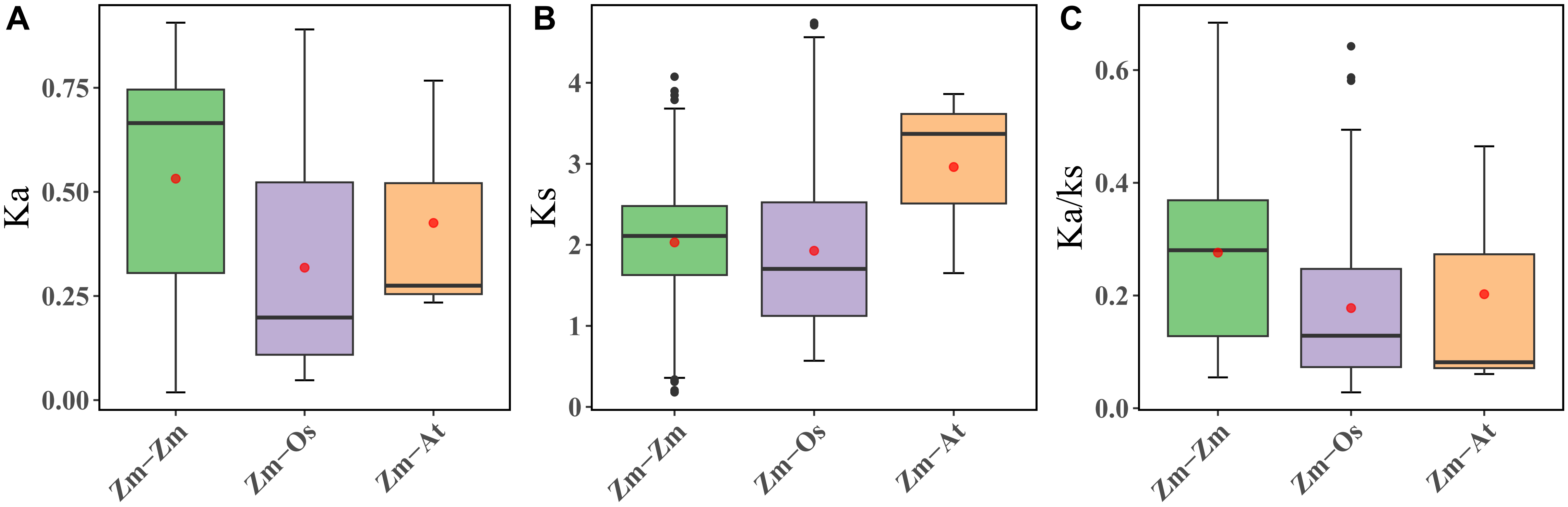

Figure S1. The duplication among rice, Arabidopsis and maize. Average values of Ka, Ks, and Ka/Ks , respectively, of duplicated genes. The horizontal axes in stand for the duplication between maize and Arabidopsis (Zm–At) and Rice (Zm-Os).
