# Supplementary material for: Genome-wide identification and transcriptome profiling expression analysis of the U-box E3 ubiquitin ligase gene family related to abiotic stress in maize (Zea mays L.)
Source: BMC Genomics. 2024 Feb 1;25:132. doi: 10.1186/s12864-024-10040-8 (PMC10832145; doi:10.1186/s12864-024-10040-8)
Supplement: Supplementary file 2 — Additional file 2. [file 12864_2024_10040_MOESM2_ESM.pdf]

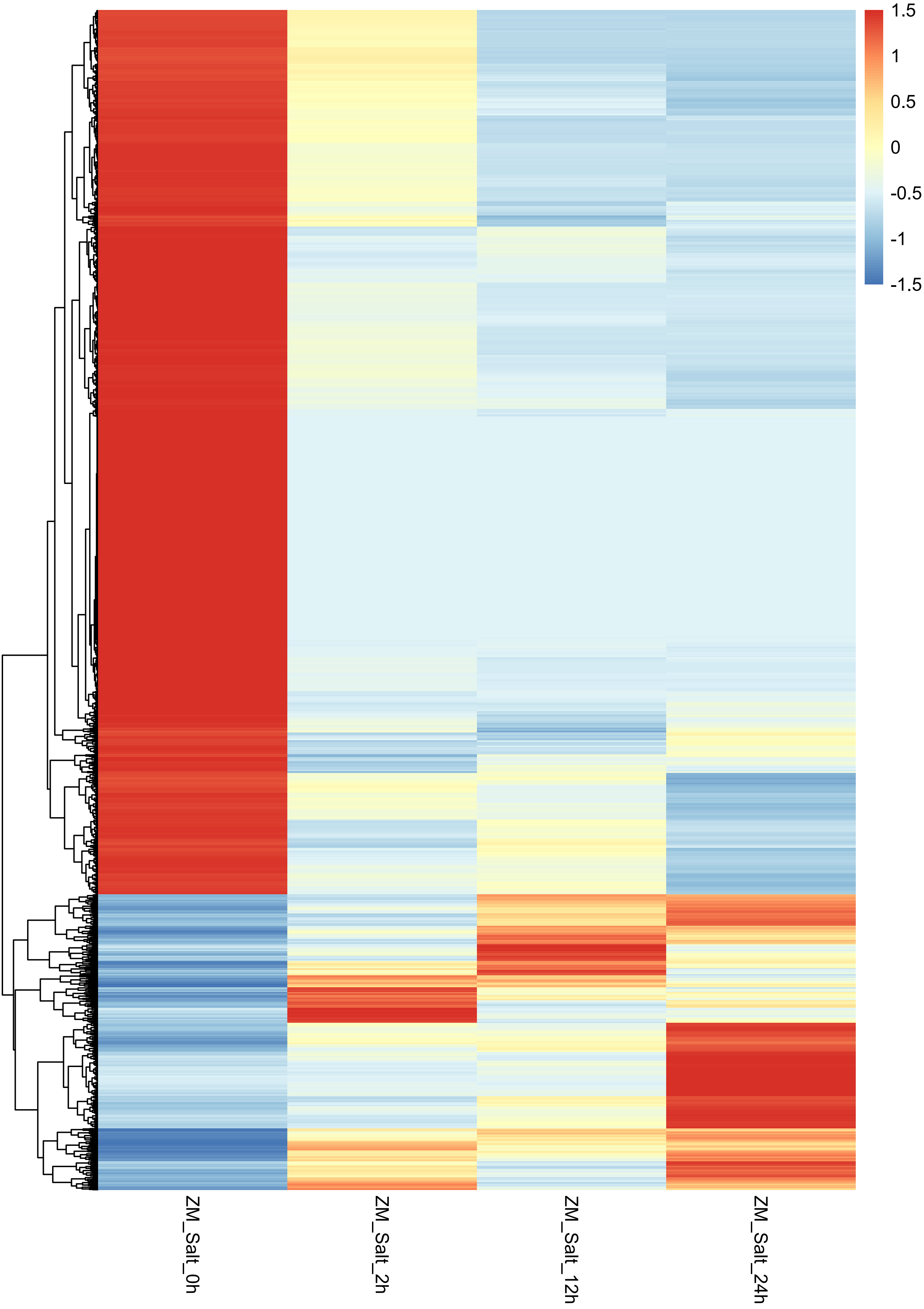

Figure S2. All DEGs responsive to salt stress. The *ZmPUBs* were analyzed for differential expression at 0, 2, 12, and 24 hours after salt treatment. High expression is depicted in red, while low expression is indicated in blue. Various genes showed distinct expression patterns both prior to and following the salt stress treatment.
