# Supplementary material for: Genome-wide identification and transcriptome profiling expression analysis of the U-box E3 ubiquitin ligase gene family related to abiotic stress in maize (Zea mays L.)
Source: BMC Genomics. 2024 Feb 1;25:132. doi: 10.1186/s12864-024-10040-8 (PMC10832145; doi:10.1186/s12864-024-10040-8)
Supplement: Supplementary file 3 — Additional file 3. [file 12864_2024_10040_MOESM3_ESM.pdf]

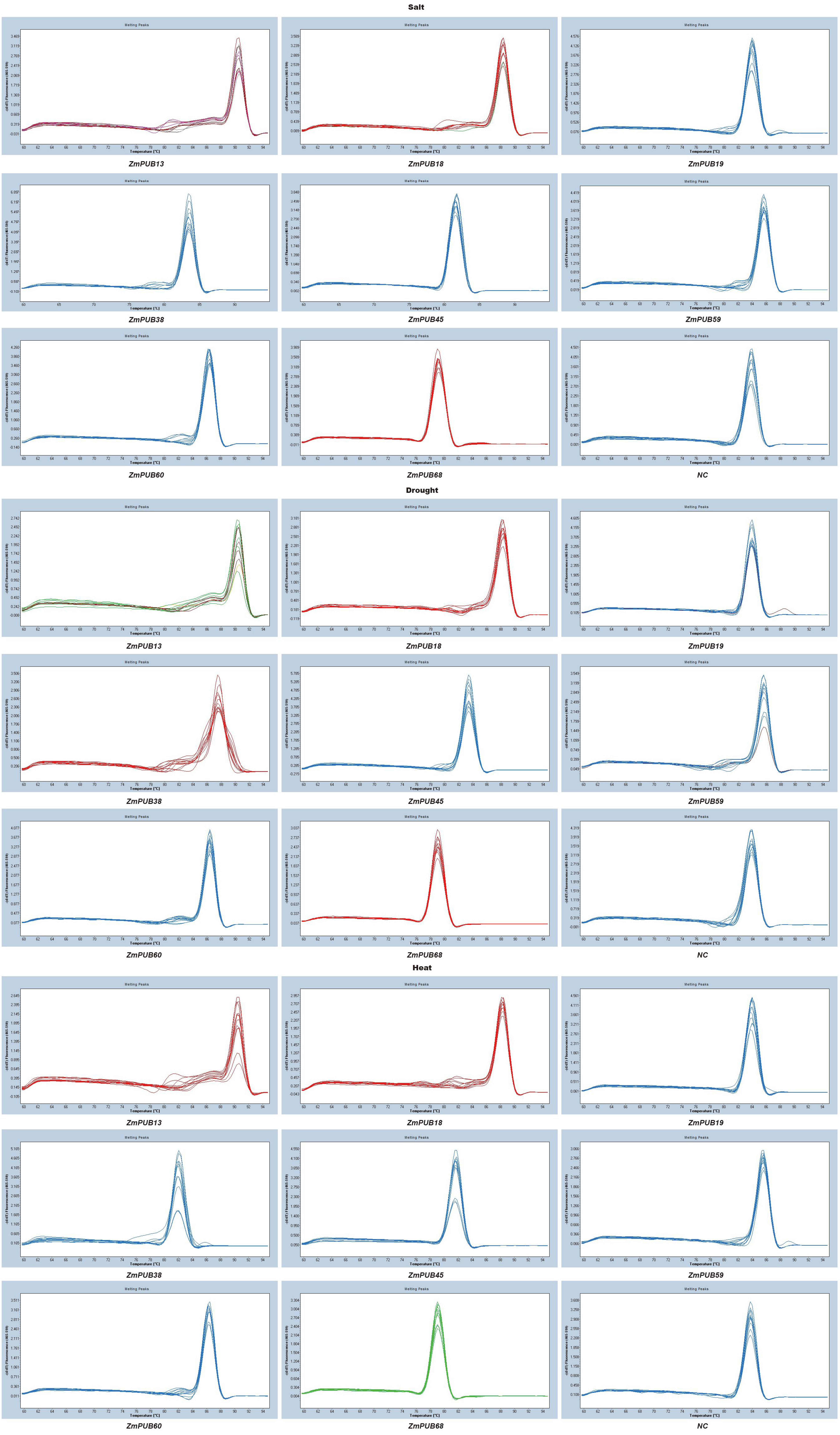


**Figure S3. Peak plot of qRT-PCR.** The peak plot of qRT-PCR for *ZmPUB13, ZmPUB18, ZmPUB19, ZmPUB38, ZmPUB45, ZmPUB59, ZmPUB60, ZmPUB68,* and NC. The melting curves of qPCR analysis for these genes all exhibit single peaks, indicating good specificity.
